# Macrophage-derived extracellular vesicle-packaged WNTs rescue intestinal stem cells and enhance survival after radiation injury

**DOI:** 10.1038/ncomms13096

**Published:** 2016-10-13

**Authors:** Subhrajit Saha, Evelyn Aranda, Yoku Hayakawa, Payel Bhanja, Safinur Atay, N Patrik Brodin, Jiufeng Li, Samuel Asfaha, Laibin Liu, Yagnesh Tailor, Jinghang Zhang, Andrew K. Godwin, Wolfgang A. Tome, Timothy C. Wang, Chandan Guha, Jeffrey W. Pollard

**Affiliations:** 1 Department of Radiation Oncology, Albert Einstein College of Medicine & Montefiore Medical Center, Bronx, New York 10461, USA; 2 Department of Developmental and Molecular Biology, Albert Einstein College of Medicine, Bronx, New York 10461, USA; 3 Department of Medicine, Division of Digestive and Liver Diseases, Irving Cancer Research Center, Columbia University, New York, New York 10032, USA; 4 Department of Pathology and Laboratory Medicine, University of Kansas Medical Center, Kansas City, Kansas 66160, USA; 5 Department of Microbiology and Immunology, Albert Einstein College of Medicine, Bronx, New York, 10461, USA; 6 Department of Pathology, Albert Einstein College of Medicine, Bronx, New York 10461, USA; 7 MRC Centre for Reproductive Health, University of Edinburgh, Edinburgh EH16 4TJ, UK

## Abstract

WNT/β-catenin signalling is crucial for intestinal homoeostasis. The intestinal epithelium and stroma are the major source of WNT ligands but their origin and role in intestinal stem cell (ISC) and epithelial repair remains unknown. Macrophages are a major constituent of the intestinal stroma. Here, we analyse the role of macrophage-derived WNT in intestinal repair in mice by inhibiting their release using a macrophage-restricted ablation of Porcupine, a gene essential for WNT synthesis. Such *Porcn*-depleted mice have normal intestinal morphology but are hypersensitive to radiation injury in the intestine compared with wild-type (WT) littermates. *Porcn*-null mice are rescued from radiation lethality by treatment with WT but not *Porcn*-null bone marrow macrophage-conditioned medium (CM). Depletion of extracellular vesicles (EV) from the macrophage CM removes WNT function and its ability to rescue ISCs from radiation lethality. Therefore macrophage-derived EV-packaged WNTs are essential for regenerative response of intestine against radiation.

## Abstract (web summary)

The intestinal stroma secretes WNT ligands but the role of WNT in intestinal repair is unclear. Here, the authors show that when WNT synthesis is ablated from stromal macrophages, the intestine morphology is normal but hypersensitive to radiation injury, implicating macrophage-derived WNT in intestinal repair.

## Graphical abstract

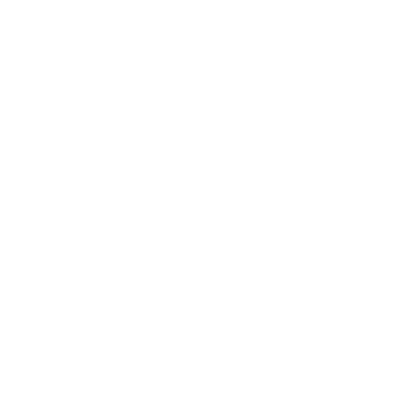

Intestinal epithelial homoeostasis depends on the signalling crosstalk between the crypt intestinal stem cells (ISC) and the surrounding niche, including the intestinal subepithelial myofibroblasts, endothelial cells and macrophages. The cells in the ISC niche provide critical growth factor/signals for ISC regeneration and intestinal homoeostasis^1^. We have recently reported that the radiation-induced gastrointestinal syndrome (RIGS) results from a combination of radiation-induced loss of crypt progenitors and stromal cells along with aberrant signalling in the ISC niches^2^. Transplantation of bone marrow-derived adherent stromal cells accelerates the restitution of the irradiated ISC niche, promotes ISC regeneration and improves survival from RIGS, following whole-body irradiation (WBI) in C57Bl/6 mice. Transplantation of the bone marrow-derived adherent stromal cell population depleted of all CD11b^+ve^ myeloid cells failed to rescue mice from lethal radiation injury^2^. However, the specific role of bone marrow-derived mature macrophages that are CD11b^+ve^ against lethal radiation injury in the intestine has not been studied.

Evidence in support of a key role for macrophages in crypt regeneration comes from studies where depletion of host macrophages with liposome-encapsulated clodronate resulted in poor survival after irradiation^2^. Macrophages play an important role in coordinating signals from gut microbes and injured epithelium, and thereby transmit regenerative signals to ISC^3^,^4^. Our previous studies have shown that TLR-mediated activation of macrophages followed by transplantation could ameliorate intestinal injury^5^. Moreover, in a mouse model of colitis, it has been shown TLR-mediated activation of colonic macrophages is critical for regeneration of colonic progenitors^4^. Nevertheless, while these earlier studies have outlined a role for macrophages in promoting intestinal regeneration, the effect of macrophages on survival and proliferation of ISCs and the nature of the signals that are transmitted has not been elucidated.

Wnt/β-catenin signalling plays a major role in ISC self-renewal and proliferation and thereby maintenance of intestinal homoeostasis^6^,^7^. WNT ligands bind to LRP5/6 and Frizzled co-receptors present on epithelial crypt cells, leading to β-catenin stabilization and nuclear translocation^8^ where it binds to the nuclear transcription factor TCF4 to drive a gene-expression programme that supports stem cell maintenance, proliferation and differentiation. Activation of WNT/β-catenin signalling is also crucial for crypt regeneration following injury. Several reports, have demonstrated that Rspondin 1 (RSPO1), an ISC growth factor and LGR5 receptor agonist, activates WNT/β-catenin pathway to repair and regenerate the intestine following chemo-radiation-induced injury^9^,^10^,^11^,^12^. Systemic administration of DKK1, a negative regulator WNT/β-catenin pathway, impairs the RSPO1-induced intestinal regeneration^13^.

WNTs are evolutionarily conserved, cysteine-rich glycoproteins capable of functioning in both a paracrine and autocrine manner. Epithelial and stromal cells of the small intestine express 19 different *Wnt* genes^7^. These WNT ligands are involved in various physiological processes including injury repair, innate and adaptive immunity, and intermediary metabolism^14^,^15^,^16^. However, the overall importance of secreted WNTs in proliferation and self-renewal of ISCs remains unknown. Some reports demonstrated that ablation of WNTs in the mouse intestine could not inhibit crypt stem cell proliferation *in vivo*^14^,^17^. WNT secretion can be inhibited by deletion of *Porcn*, an indispensable part of the core WNT ligand secretion machinery^18^,^19^. PORCN is a trans-membrane endoplasmic reticulum O-acyl transferase^20^ encoded by a single copy gene on the X chromosome and is involved in palmitoleation of all WNT molecules synthesized in the cell^21^,^22^,^23^. Palmitoleation is an essential process for post-translational modification of WNT that is needed for binding of WNTs with wingless, an integral membrane carrier protein that is essential for the secretion of all known vertebrate WNTs and their binding to Frizzled receptors^18^,^24^,^25^. It has been reported that mice deficient in all intestinal epithelial WNTs due to deletion of the gene Porcupine (*Porcn*) by Villin-Cre have normal ISC proliferation and homoeostasis *in vivo*^1^. However inhibition of WNT release from both epithelial and stromal sources by pharmacological inhibition of Porcupine showed decrease in epithelial proliferation. Moreover it was noted that global pharmacologic but not epithelial-specific inhibition of *Porcn* significantly increased the radio-sensitivity of intestine^1^. These observations clearly suggest the importance of stromal cell-derived WNTs in intestinal homoeostasis and regeneration.

Macrophages maybe one of the sources of stromal WNTs in the crypt as in other contexts macrophage-derived WNTs have been reported to have a significant role in tissue repair and regeneration^26^,^27^. For example macrophage-derived WNT7b was shown to be crucial for epithelial regeneration in response to kidney injury^28^ and for hepatocyte regeneration in the liver^29^. In addition, macrophage-derived WNTs have been shown to affect blood vessel formation by regulating VEGF and angiopoietin signalling in vascular endothelial cells^27^.

In this study to define the role of macrophage-derived WNTs in intestinal repair and regeneration following radiation injury, we used a genetic approach to block WNT release from macrophages by ablating a floxed allele of *Porcn* with a mononuclear phagocyte restricted cre-recombinase expressed from the colony-stimulating factor-1 receptor (*Csf1r*) promoter (*Csf1r.icre* (ref. 30). Using this strategy we found that inhibition of macrophage-derived WNTs exacerbated RIGS, impaired recovery from radiation injury with the loss of Lgr5^+ve^ crypt base columnar cells (CBCs) and decreased survival. Treatment with wild-type (WT) bone marrow macrophage (BMMφ)-conditioned medium (CM) or WNT-deficient Porcupine-null BMMφ CM supplemented with exogenous WNT ligands rescued the crypt epithelium from radiation toxicity. We have also demonstrated that macrophage-derived WNTs are expressed by BMMΦ and secreted through extracellular vesicle (EV)^31^ and therefore treatment with purified EVs could rescue crypt cells from radiation toxicity. This radioprotective activity was lost on EV depletion from BMMΦ CM indicating the ligands are secreted in EVs. However, deletion of *Porcn* in macrophages in non-irradiated mice did not show any obvious phenotype in the intestine indicating that the macrophage-derived WNT activity is specific for repair and regeneration. Taken together, these observations indicate that macrophage-derived EV-packaged WNTs are critical for regenerative response of intestine following injury.

## Results

### *Porcn* deletion in Mφ sensitizes gut to lethal doses of WBI

To assess the role of macrophage-derived WNT in intestinal regeneration, mice carrying a floxed allele of *Porcn*^21^ were crossed to *Csf1r.iCre* male mice^30^ to generate *Csf1r.iCre*;*Porcn*^*fl/fl*^ mice that will have a deletion of *Porcn* restricted to macrophages. Analysis of BMMφ-derived DNA from the *Porcn*^*fl/fl*^ mice with or without cre showed efficient ablation of the floxed allele in the presence of *Csf1r.icre* with loss of the 248 bp floxed allele and generation of a 386 bp recombined allele (Supplementary Fig. 1a). Macrophage-specific deletion of *Porcn* was confirmed at the expression level by PCR of DNA isolated from BMMφ, pulmonary and crypt epithelial cells and hepatocytes of *Csf1r.iCre*;*Porcn*^*fl/fl*^ mice. The presence of porcupine floxed allele was detected in all the tissue samples except in the targeted BMMφ (Supplementary Fig. 1b). Quantitative real-time (QPCR) PCR using both RNA and DNA of the cre-containing versus cre-negative BMMφ indicated ∼80% allele ablation typical for floxed alleles (Supplementary Fig. 1c,d respectively). A TCF/LEF (TOPFLASH) reporter assay^32^ was used to measure WNT/β-catenin activity and this assay demonstrated that CM from *Csf1r.iCre*;*Porcn*^*fl/fl*^ mice BMMφ failed to activate the β-catenin pathway whereas Cre-negative littermates referred to as WT BMMφ showed activity (Supplementary Fig. 1e). Pharmacological inhibition of Porcupine in macrophages using C59 a porcupine inhibitor^1^ also abolished WNT activity in BMMφ CM as determined by the TOPFLASH reporter assay. In contrast no activity was seen in the control FOPFLASH assay (mutant TCF/LEF reporter) indicating specificity for WNTs in the TOPFLASH assay. These data indicate that ablation of the *Porcn* floxed allele causes the absence of macrophage-derived WNT in BMMφ CM and is similar to the pharmacological inhibition of Porcupine in macrophages. Since the population size of intestinal pericryptal macrophages is not sufficient to culture and obtain CM throughout the present study we have used BMMφ CM from WT and *Porcn*-deficient mice to determine the functional role of macrophage-derived WNTs.

Before determining the effect on radiation-induced repair, we assessed whether genetic ablation of *Porcn* in macrophages had significant consequences for mice and their intestinal development. *Csf1r.iCre*;*Porcn*^*fl/fl*^ mice appeared phenotypically normal, having similar lifespans (followed up to 18 months of age), and intestinal morphology (Supplementary Fig. 2a), compared with WT. The total number of intestinal macrophages (CD11b^+ve^ CX3CR1^+ve^ F480^+ve^; Supplementary Fig. 2b) were similar in *Csf1r.iCre*;*Porcn*^*fl/fl*^ and WT with/without irradiation indicating that *Porcn* deletion does not influence the population size of intestinal macrophages.

**Figure 1:**
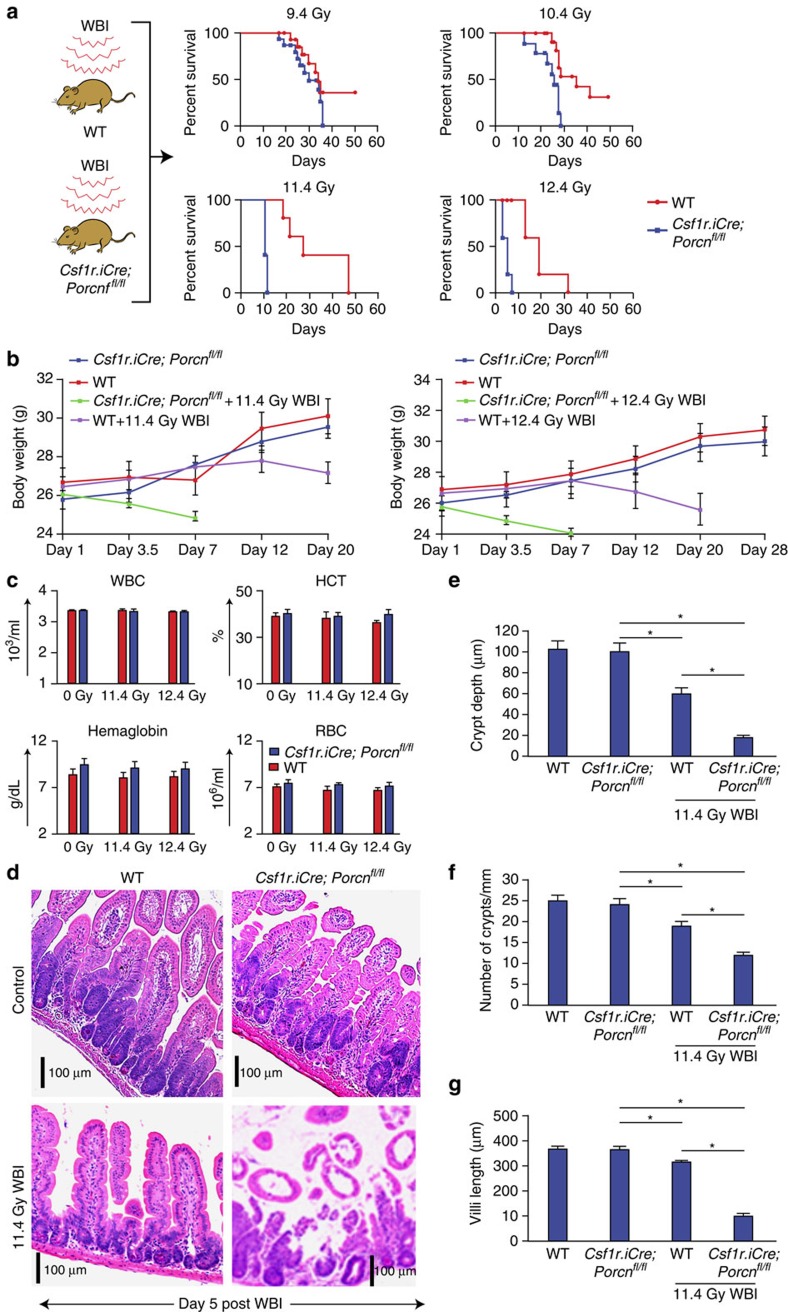
Deletion of *Porcn* in macrophages radio-sensitizes mice to lethal doses of WBI. (**a**) Kaplan–Meier survival analysis of *Csf1r.iCre*;*Porcn*^*fl/fl*^ and WT mice exposed to WBI (9.4–12.4 Gy). *Csf1r.iCre*;*Porcn*^*fl/fl*^ mice show reduced survival following lethal doses of WBI (11.4–12.4 Gy WBI) with 100% mortality within 7–12 days of radiation exposure, compared with WT that have 60% survival beyond 15 days post WBI (*P*<0.0001, *P*<0.004 Log-rank (Mantel–Cox) test; *n*=15 per group). No significant survival difference between *Csf1r.iCre*;*Porcn*^*fl/fl*^ or WT littermate mice was observed with the irradiation dose of 9.4–10.4 Gy WBI (*n*=15 per group). (**b**) Body weight of mice at post irradiation time points (11.4 Gy and 12.4 Gy WBI). (**c**) Complete blood count (CBC) analysis. CBC measurements for *Csf1r.iCre*;*Porcn*^*fl/fl*^ and WT mice exposed to 0 Gy/11.4 Gy/12.4 Gy WBI. Blood samples were drawn at 5 days post irradiation (*n*=3 per group). (**d**) HE staining of jejunum section from *Csf1r.iCre*;*Porcn*^*fl/fl*^ and WT mice exposed to 0 Gy/11.4 Gy WBI at day 5 post irradiation. No significant differences in crypt villus structure were noted in non-irradiated *Csf1r.iCre*;*Porcn*^*fl/fl*^ mice compared with WT mice. Note, shortening of crypt depth as well as loss of crypts in *Csf1r.iCre*;*Porcn*^*fl/fl*^ mice exposed to irradiation (*n*=3 per group) compared with WT mice. (**e**) Histogram demonstrating crypt depth (μM) from jejunal sections of *Csf1r.iCre*;*Porcn*^*fl/fl*^ and WT mice exposed to 0 or 11.4 Gy WBI. Both WT and *Csf1r.iCre*;*Porcn*^*fl/fl*^ mice exposed to irradiation showed reduction in crypt depth at day 5 post irradiation compared with un-irradiated control WT mice *P<4.66E−05, *Csf1r.iCre*;*Porcn*^*fl/fl*^ mice *P<9.63E−08. However, loss of crypt depth was significantly greater in *Csf1r.iCre*;*Porcn*^*fl/fl*^ mice compared with WT mice *P<8.84E−07 unpaired *t*-test, two-tailed. (**f**) Histogram demonstrating the number of crypts mm^−1^ from jejunal section of *Csf1r.iCre*;*Porcn*^*fl/fl*^ and WT mice exposed to 0 or 11.4 Gy WBI. Both WT and *Csf1r.iCre*;*Porcn*^*fl/fl*^ mice exposed to irradiation showed reduction in crypt number at day 5 post irradiation compared with un-irradiated control WT mice *P<0.0008, *Csf1r.iCre*;*Porcn*^*fl/fl*^ mice *P<1E−08. However, loss of crypt was significantly greater in *Csf1r.iCre*;*Porcn*^*fl/fl*^ mice compared with WT mice *P<9.79E−09 unpaired *t*-test, two-tailed. (**g**) Histogram demonstrating villi length from jejunal section of *Csf1r.iCre*;*Porcn*^*fl/fl*^ and WT mice exposed to 0 or 11.4 Gy WBI. Both WT and *Csf1r.iCre*;*Porcn*^*fl/fl*^ mice exposed to irradiation showed reduction in crypt number at day 5 post irradiation compared with un-irradiated control WT mice*P<0.0006, *Csf1r.iCre*;*Porcn*^*fl/fl*^ mice *P<8.9E−08. However, loss of villi length was significantly greater in *Csf1r.iCre*;*Porcn*^*fl/fl*^ mice compared with WT mice *P<7.98E−09 unpaired *t*-test, two-tailed.

Mortality from acute radiation syndrome results from dose-dependent radiation injury to various organs^2^,^9^,^33^. Female *Csf1r.iCre*;*Porcn*^*fl/fl*^ and WT mice were exposed to WBI and observed over 60 days for survival. No significant survival difference between *Csf1r.iCre*;*Porcn*^*fl/fl*^ or WT mice was observed with the dose of 9.4–10.4 Gy (*P*<0.9 and *P*<0.07, respectively). However, with higher radiation doses (11.4–12.4 Gy WBI) *Csf1r.iCre*;*Porcn*^*fl/fl*^ mice demonstrated significantly reduced survival, with 100% mortality within 7–12 days of WBI, compared with the WT cohort having 60% survival beyond 15 days post WBI (Fig. 1a, *P*<0.0001, *P*<0.004 Log-rank (Mantel–Cox) test).

The time to death after lethal radiation from haematopoietic failure and RIGS is dose-dependent. Moreover, ionizing radiation induces GI syndrome independent of bone marrow syndrome^34^. A WBI exposure of >10.4 Gy within 5–14 days results in a characteristic RIGS, comprising of diarrhoea, weight loss and death. However, mortality at later time points (for example, 25–30 days) post irradiation (<10.4 Gy) is attributed to the radiation-induced haematopoietic syndrome. In the present study, there was 100% mortality in *Csf1r.iCre*;*Porcn*^*fl/fl*^ mice within 7–12 days of WBI of 11.4–12.4 Gy (Fig. 1a), suggesting their susceptibility to RIGS compared with the WT littermates. This conclusion was also supported by the observation of a reduction of body weight in the *Csf1r.iCre*;*Porcn*^*fl/fl*^ mice treated with 11.4 Gy by day 7 that was not observed in the WT littermates (Fig. 1b). However, the onset of mortality was at much later time points in *Csf1r.iCre*;*Porcn*^*fl/fl*^ mice or their WT littermates exposed to ≤10.4 Gy (Fig. 1a) indicating that RIGS may not be the primary cause of death in these animals. These results further indicate that *Csf1r.iCre*;*Porcn*^*fl/fl*^ mice were more sensitive than WT controls to a radiation dose level that primarily induces RIGS.

Consistent with the effect of macrophage WNT loss enhancing RIGS and not effecting haematopoiesis, was that blood counts designed to assess haematopoietic syndrome did not show any differences among *Csf1r.iCre*;*Porcn*^*fl/fl*^ mice and their WT littermate exposed to radiation (11.4–12.4 Gy; Fig. 1c). However, the histology of intestinal (jejunal) sections from irradiated mice showed more loss of crypt villus structure in *Csf1r.iCre*;*Porcn*^*fl/fl*^ mice with significant reduction in Crypt depth, villi length and number of crypts compared with their WT controls (Crypt depth *P<8.84E−07, villi length *P<7.98E−08 and number of crypt *P<9.79E−09 unpaired *t*-test, two-tailed; Fig. 1d–g).

### *Porcn* deletion in Mφ sensitize gut to Abdominal Irradiation

**Figure 2:**
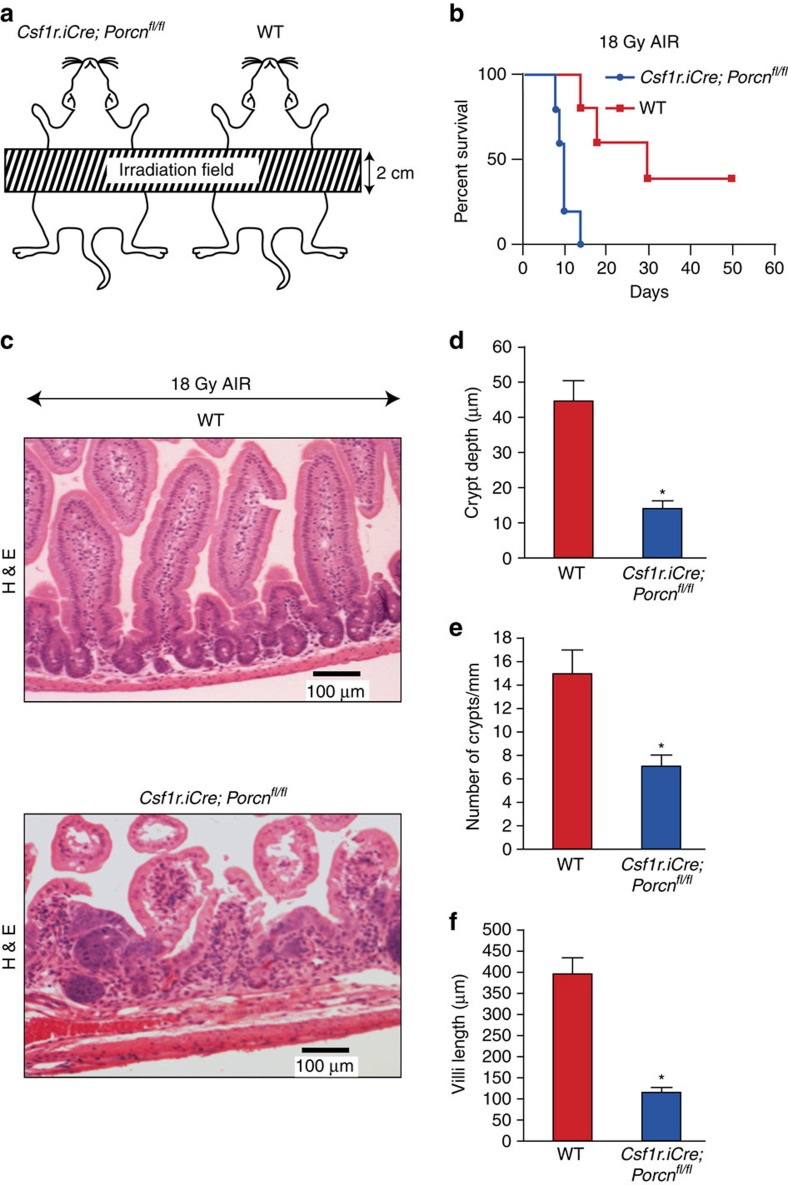
Deletion of *Porcn* in macrophages sensitizes mice against lethal dose of AIR. (**a**) Schematic diagram demonstrating the AIR exposure field for *Csf1r.iCre*;*Porcn*^*fl/fl*^ and WT mice. A 2 cm area of the mice containing the GI was irradiated (irradiation field), thus shielding the upper thorax, head and neck as well as lower and upper extremities, protecting a significant portion of the bone marrow, thus inducing predominantly RIGS. (**b**) Kaplan–Meier survival analysis. *Csf1r.iCre*;*Porcn*^*fl/fl*^ mice have reduced survival against a lethal dose (18 Gy) of abdominal radiation compared with WT mice (*n*=10 per group; *P*<0.009 Log-rank (Mantel–Cox) test). (**c**) HE staining of jejunum sections from *Csf1r.iCre*;*Porcn*^*fl/fl*^ and WT mice exposed to 18 Gy AIR. Mice were killed and jejunum was collected at day 5 post irradiation. *Csf1r.iCre*;*Porcn*^*fl/fl*^ mice showed more villi denudation and crypt loss compared with WT littermate mice at day 5 post irradiation (*n*=3 per group). (**d**) Histogram showing crypt depth (μM) in jejunal sections of *Csf1r.iCre*;*Porcn*^*fl/fl*^ and WT mice exposed to 0 Gy or 18 Gy AIR. *Csf1r.iCre*;*Porcn*^*fl/fl*^ mice exposed to AIR had significantly higher reduction in crypt depth compared with WT *P<9.64E−08 unpaired *t*-test, two-tailed. (**e**) Histogram showing number of crypts mm^−1^ in jejunal sections of *Csf1r.iCre*;*Porcn*^*fl/fl*^ and WT mice exposed to 0 Gy or 18 Gy AIR. *Csf1r.iCre*;*Porcn*^*fl/fl*^ mice exposed to AIR has significantly higher reduction in crypt number compared with WT *P<8.77E−09 unpaired *t*-test, two-tailed. (**f**) Histogram showing villus length in jejunal sections of *Csf1r.iCre*;*Porcn*^*fl/fl*^ and WT mice exposed to 0 or 18 Gy AIR. *Csf1r.iCre*;*Porcn*^*fl/fl*^ mice exposed to AIR has significantly higher reduction in villi length compared with WT *P<8E−07 unpaired *t*-test, two-tailed.

Since the effects of 10.4 Gy of WBI are secondary to combined haematopoietic and RIGS, we examined the effects of conditional *Porcn* deletion in myeloid cells on isolated RIGS injury by administering escalating doses of Abdominal Irradiation (AIR) after shielding the thorax, head and neck, and extremities, thus protecting the bone marrow^2^,^9^ (Fig. 2a). A single fraction of 18–20 Gy AIR was lethal in 100% of *Csf1r.iCre*;*Porcn*^*fl/fl*^ mice by 7–12 days. In contrast, 40% of WT littermates survived beyond 20 days post 18 Gy AIR (*P*<0.009, Log-rank (Mantel–Cox) test; Fig. 2b). Histopathology of jejunal sections at day 5 post AIR showed greater damage of the crypt villus structure with significant reduction in crypt depth (*P*<9.64E−08), villi length (*P*<7.8-E07) and number of crypts (*P*<8.77E−09 (unpaired *t*-test, two-tailed) in *Csf1r.iCre*;*Porcn*^*fl/fl*^ mice compared with their WT littermate (Fig. 2c–f). As the major part of the bone marrow was shielded, bone marrow was not severely damaged by AIR^2^, and therefore the observed mortality of the *Csf1r.iCre*;*Porcn*^*fl/fl*^ mice was due to RIGS. Altogether these data clearly indicate that inhibition of myeloid-derived WNT release in *Csf1r.iCre*;*Porcn*^*fl/fl*^ mice primarily increases gastrointestinal radio-sensitivity and facilitates the onset of RIGS.

### WT but not *Porcn*-null BMMΦ CM treatment inhibits RIGS

**Figure 3:**
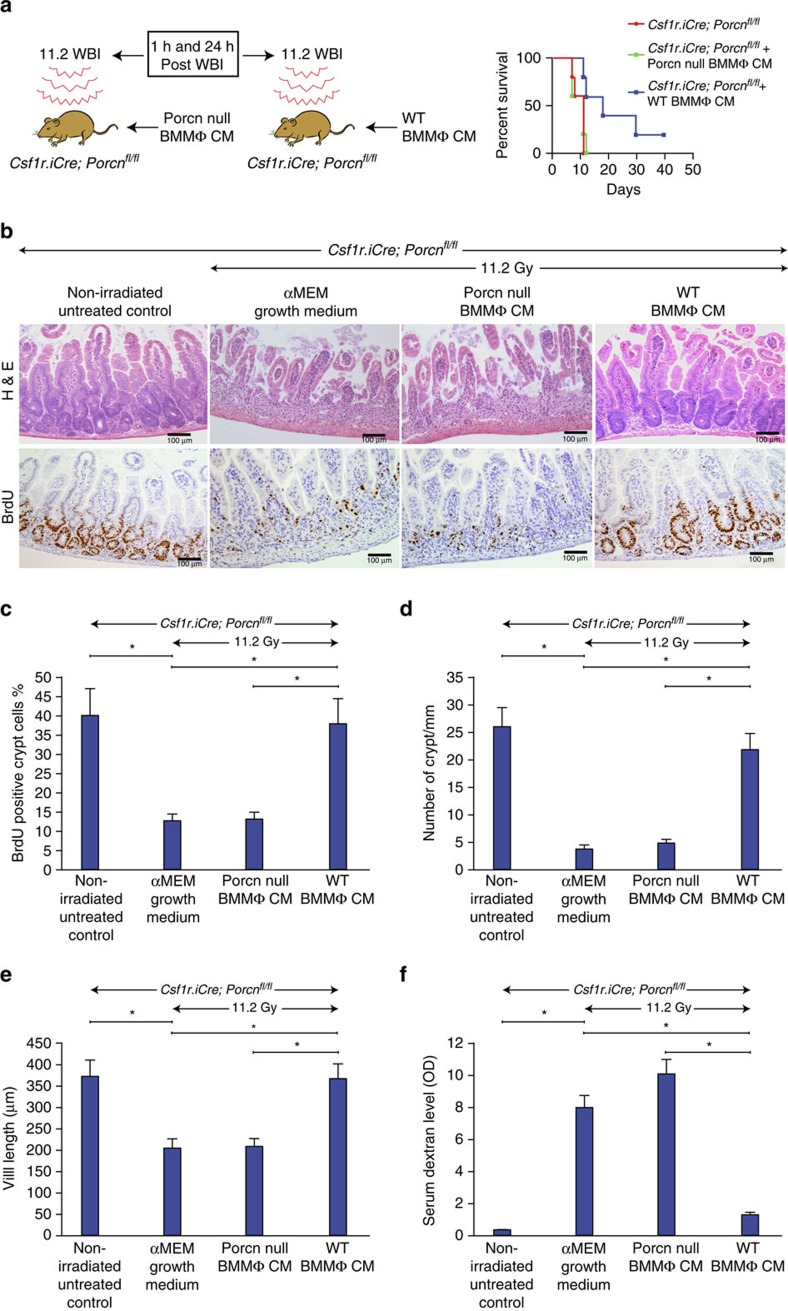
CM from WT but not from *Porcn*-null BMMΦ inhibits RIGS in *Csf1r.iCre*;*Porcn*^*fl/fl*^ mice exposed to lethal dose of WBI. (**a**) Experimental design and Kaplan–Meier survival analysis of *Csf1r.iCre*;*Porcn*^*fl/fl*^ mice (*n*=10 per group) receiving CM (i.v.) derived from WT/*Porcn*-null BMMΦ at 1 and 48 h post WBI (11.2 Gy). Mice receiving WT BMMΦ CM showed 40% survival (*P*<0.003 Log-rank (Mantel–Cox) test) beyond 25 days compared with mice receiving *Porcn*-null BMMΦ CM or untreated mice where 100% of mice died within 12 days after irradiation. (**b**) Representative HE and BrdU immunohistochemistry of mice jejunal sections. Note, restitution on crypt villus structure with the increase in crypt cell proliferation in *Csf1r.iCre*;*Porcn*^*fl/fl*^ mice receiving WT BMMΦ CM compared with *Porcn*-null BMMΦ CM treatment. (**c**) The proliferation rate was calculated as the percentage of BrdU-positive cells over the total number of cells in each crypt and displayed as bar diagrams. Crypt cell proliferation rate in irradiated mice: WT BMMΦ CM versus *Porcn*-null BMMΦ CM treatment group *P<2.21E−07 (*n*=5 per group), WT BMMΦ CM versus αMEM growth medium treatment group *P<2.69E−07 (*n*=5 per group; unpaired *t*-test, two-tailed). (**d**) Histogram showing number of crypts mm^−1^ in jejunal sections of *Csf1r.iCre*;*Porcn*^*fl/fl*^ mice. Irradiated *Csf1r.iCre*;*Porcn*^*fl/fl*^ mice receiving WT BMMΦ CM showed less crypt loss compared with mice receiving *Porcn*-null BMMΦ CM or αMEM growth medium (*P<6.86E−09 and *P<6.74E−08 unpaired *t*-test, two-tailed). (**e**) Histogram showing villus length in jejunal sections of *Csf1r.iCre*;*Porcn*^*fl/fl*^ mice. Irradiated *Csf1r.iCre*;*Porcn*^*fl/fl*^ mice receiving WT BMMΦ CM showed less reduction in villi length compared with mice receiving *Porcn*-null BMMΦ CM or αMEM growth medium (*P<3.74E−06 and *P<3.60E−06 unpaired *t*-test, two-tailed). (**f**) Histogram demonstrating serum dextran level in *Csf1r.iCre*;*Porcn*^*fl/fl*^ mice. Mice receiving WT BMMΦ CM showed a lower serum dextran level thereby suggesting restitution of epithelial integrity compared with mice receiving *Porcn*-null BMMΦ CM or αMEM growth medium (*P<0.006 and *P<0.009 respectively; *n*=3 per group). Untreated mice also showed a lower serum dextran level compared with irradiated mice receiving αMEM growth medium (*P*<0.003) or *Porcn*-null BMMΦ CM (*P*<0.001; unpaired *t*-test, two-tailed).

Since WT BMMΦ CM had shown higher WNT activity compared with CM from porcupine-null BMMΦ (Supplementary Fig. 1e), we examined whether BMMΦ CM could rescue *Csf1r.iCre*;*Porcn*^*fl/fl*^ mice from RIGS. *Csf1r.iCre*;*Porcn*^*fl/fl*^ mice exposed to WBI (11.2 Gy) were treated with CM (i.v.) derived from WT or *Porcn*-null BMMΦ at 1 h and 24 h post WBI. Animals only exposed to 11.2 Gy WBI or receiving porcupine-null BMMΦ CM after radiation died within 12 days of radiation exposure (Fig. 3a) with characteristic signs and symptoms of RIGS, including diarrhoea, black stools and weight loss. In contrast, animals that received WT BMMΦ CM had well-formed stools, maintained body weight and had 40% survival beyond 20 days (*P*<0.01, Log-rank (Mantel–Cox) test; Fig. 3a).

Histopathological analysis of jejunal section at 3.5 days post WBI showed crypt depletion and a decrease in crypt regeneration followed by villi denudation in *Csf1r.iCre*;*Porcn*^*fl/fl*^ mice (Fig. 3b,d,e). Treatment with *Porcn*-null BMMΦ CM failed to rescue the crypt villus structure following irradiation (Fig. 3b,d,e). However, CM from WT BMMΦ improved the overall crypt villus architecture in irradiated *Csf1r.iCre*;*Porcn*^*fl/fl*^ mice, with an increase in number of crypts and preserved villous length (Fig. 3b,d,e). The percentage of the BrdU^+ve^ crypt epithelial cells synthesizing DNA was significantly enhanced in WT cohort at 3.5 days post irradiation (*P*<2.21E−07 unpaired *t*-test, two-tailed; Fig. 3b,c). However, treatment with CM from WT or *Porcn*-null BMMΦ in non-irradiated mice did not induce any changes in crypt villus morphology and BrdU incorporation (Supplementary Fig. 3).

Since dextran is unable to cross the GI epithelia unless it is compromised, dextran in the blood is a good indicator of epithelial damage^35^, blood FITC-dextran levels were measured at 4 h after gavage. Treatment with WT BMMΦ CM significantly reduced the FITC-dextran uptake in the blood stream in irradiated *Csf1r.iCre*;*Porcn*^*fl/fl*^ mice compared with mice receiving *Porcn*-null BMMΦ CM (*P*<0.007 unpaired *t*-test, two-tailed; Fig. 3f). These data indicate restitution of intestinal epithelial integrity by BMMΦ CM.

**Figure 4:**
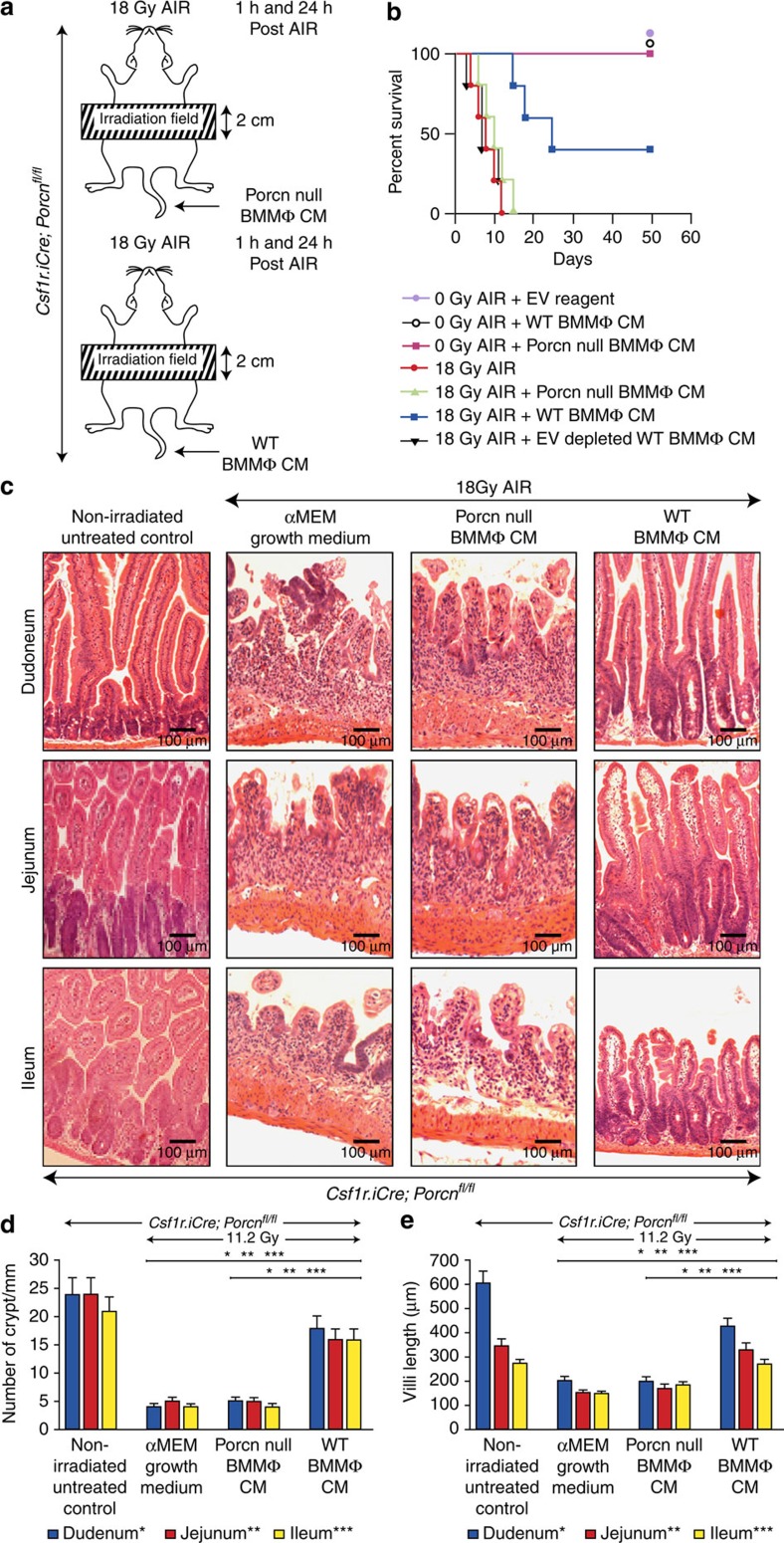
WNTs in BMMΦ CM are required to inhibit RIGS in *Csf1r.iCre*;*Porcn*^*fl/fl*^ mice exposed to AIR. (**a**) Experimental design for partial body irradiation. Mice exposed to AIR were treated with WT or *Porcn*-null BMMΦ CM at 1 and 24 h post exposure. (**b**) Kaplan–Meier survival analysis of *Csf1r.iCre*;*Porcn*^*fl/fl*^ mice (*n*=10 per group) receiving CM (500 μl per mice i.v.) derived from WT or *Porcn*-null BMMΦ at 1 h and 48 h post AIR (18 Gy). Mice receiving WT BMMΦ CM showed 60% survival beyond 20 days compared with mice receiving *Porcn*-null BMMΦ CM or EV-depleted WT BMMΦ CM, where 100% of mice died within 12 days after irradiation (*P*<0.002 and *P*<0.003, respectively, Log-rank (Mantel–Cox) test). Reagent used for chemical depletion of EV did not confer any toxicity to normal mice. (**c**) HE-stained representative transverse sections of duodenum, jejunum and ileum from *Csf1r.iCre*;*Porcn*^*fl/fl*^ mice (*n*=3 per group). Note, restitution on crypt villus structure in irradiated *Csf1r.iCre*;*Porcn*^*fl/fl*^ mice receiving WT BMMΦ CM. However, treatment with *Porcn*-null BMMΦ CM or αMEM growth medium showed significant loss of crypts along with villi denudation. (**d**) Histogram demonstrating number of crypts mm^−1^ in duodenal, jejuna, and illeul sections of *Csf1r.iCre*;*Porcn*^*fl/fl*^ mice. Irradiated *Csf1r.iCre*;*Porcn*^*fl/fl*^ mice receiving WT BMMΦ CM showed less crypt loss compared with mice receiving *Porcn*-null BMMΦ CM (Duodenum *P<6.86E−07, Jejunum **P<7.89E−08 and Ileum ***P<8.16E−08) or αMEM growth medium (Duodenum *P<7.92E−08, Jejunum **P<8.26E−07 and Ileum ***P<8.96E−09; unpaired *t*-test, two-tailed). (**e**) Histogram demonstrating villus length in duodenal, jejunal and illeul sections of *Csf1r.iCre*;*Porcn*^*fl/fl*^ mice. Irradiated *Csf1r.iCre*;*Porcn*^*fl/fl*^ mice receiving WT BMMΦ CM showed less reduction in villus length compared with mice receiving *Porcn*-null BMMΦ CM (Duodenum *P<3.64E−06, Jejunum **P<2.86E−06 and Ileum ***P<2.16E−05) or αMEM growth medium (Duodenum *P<4.21E−06, Jejunum **P<3.16E−08 and Ileum ***P<2.88E−05; unpaired *t*-test, two-tailed).

*Csf1r.iCre*;*Porcn*^*fl/fl*^ mice exposed to AIR (18 Gy) were rescued with the WT BMMΦ CM treatment with 60% mice surviving beyond 20 days of exposure (*P*<0.002 Log-rank (Mantel–Cox) test; Fig. 4a,b). In contrast, *Csf1r.iCre*;*Porcn*^*fl/fl*^ mice exposed to AIR and receiving *Porcn*-null BMMΦ CM died within 10–12 days post AIR similar to irradiated untreated control. Histopathological evidence clearly indicates that RIGS is the primary cause of death in *Porcn*-null BMMΦ CM-treated animals as there was a significant loss of crypt and villus denudation (Fig. 4c–e). However, irradiated mice receiving WT BMMΦ CM had a significant restitution of crypt villus structure (Fig. 4c–e). These results indicate that inhibition of BMMΦ derived WNT in the CM is critical for regenerative response against RIGS. However, further optimization of CM treatment is needed for complete recovery against RIGS induced by lethal dose of AIR.

### EV depletion reverses radio-mitigating role of WT BMMΦ CM

EVs are involved in the secretion of WNT proteins and have been shown to transmit morphogen signalling^36^. It has also been reported that depletion of EVs in cell culture supernatant reduces WNT activity^37^. Considering the hydrophobic properties of WNTs^38^,^39^, their normal short half-life in serum^37^ and their paracrine action in the BMMΦ CM treatment experiments shown here, we examined whether WNTs are secreted in EVs protecting them from degradation. We first depleted EV from WT BMMΦ CM to reduce the WNT activity and therefore ablate the radio-mitigating role of BMMΦ CM treatment. Depletion of EV from WT BMMΦ CM was confirmed by the absence of TSG101, ALIX and CD9 positive band that is diagnostic of EVs^37^ in the depleted fraction (Supplementary Fig. 4a). TCF/LEF reporter assay demonstrated that depletion of EVs removed the WNT activity from WT BMMΦ CM (Supplementary Fig. 4b) which was recovered with add-back of the purified EV fraction to the depleted fraction (Supplementary Fig. 4b). Concentration of the EV fraction was required to recover activity probably because the EV preparation was not optimized to maintain WNT activity. We also confirmed that absence of WNT activity in the depleted fraction is not due to adsorption of non-EV-packaged WNT by the EV depletion as addition of recombinant WNT6 or WNT9a (1 μg ml^−1^) before EV depletion showed WNT activity in EV-depleted fraction (Supplementary Fig. 4b).

**Figure 5:**
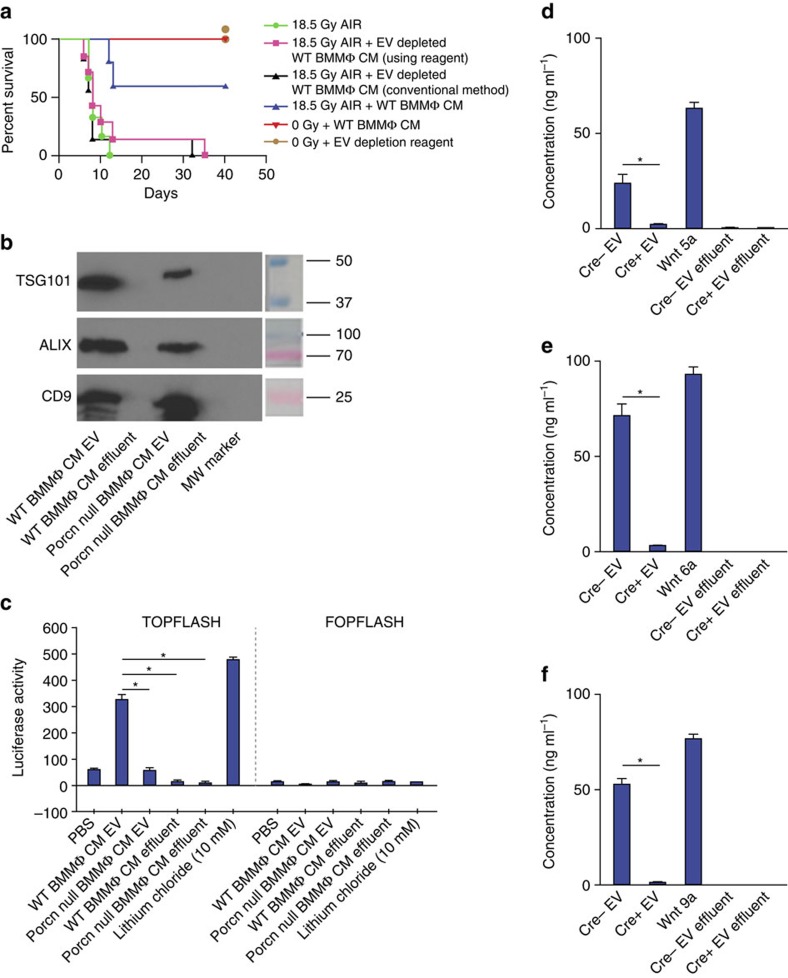
Presence of EV-packaged WNT in BMMΦ CM is critical for radio-mitigating function. (**a**) Kaplan–Meier survival analysis of WT mice (*n*=10 per group) receiving CM/EV-depleted CM (500 μl per mice i.v.) derived from WT BMMΦ at 1 h and 48 h post 18.5 Gy AIR. Mice receiving WT BMMΦ CM showed 60% survival beyond 30 days compared with mice receiving EV-depleted WT BMMΦ CM (using reagent (*P*<0.004) or conventional method (*P*<0.002)) or untreated mice (*P*<0.0009; Log-rank (Mantel–Cox) test) where 80–100% of mice died within 12 days after irradiation (*n*=10 per group). Reagent (500 μl per mice i.v.) used for chemical depletion of exosome did not confer any toxicity to normal mice. (**b**) Immunoblot to detect exosomal markers TSG101, ALIX and CD9 in EV from WT BMMΦ CM or *Porcn*-null BMMΦ CM or respective effluents. EV from WT BMMΦ CM and from *Porcn*-null BMMΦ CM showed the presence of EV markers. However, EV markers were not detected in effluents. (**c**) TCF/LEF reporter assay. HEK293 cells having TCF/LEF luciferase reporter construct were treated with EV (prepared with the conventional method) from WT or *Porcn*-null BMMΦ CM or effluents or LiCl. Treatment with EV (100 μg ml^−1^) from WT BMMΦ CM showed higher Luciferase activity compared with EV (100 μg ml^−1^) from *Porcn*-null BMMΦ CM (*P*<0.0002) and effluent from WT BMMΦ CM or *Porcn*-null BMMΦ CM (*P*<0.0001 and *P*<0.0002 respectively; unpaired *t*-test, two-tailed). (**d**–**f**) ELISA to detect WNT5a, 6 and 9a in EVs from WT or *Porcn*-null BMMΦ CM and effluents. Presence of WNT5a, WNT6 and WNT9a were detected in EVs from WT BMMΦ CM (Cre− EV) but not in EVs from *Porcn*-null BMMΦ CM (Cre+ EV; *P*<0.0002; *P*<2.78E−05 and *P*<3.26E−06 respectively; unpaired *t*-test, two-tailed). WNT5a, WNT6 and WNT9a were also absent in effluents derived from WT or *Porcn*-null BMMΦ CM. Recombinant WNT5a, WNT6 and WNT9a were used as positive control respectively.

Treatment with the EV-depleted WT BMMΦ CM could not rescue *Csf1r.iCre*;*Porcn*^*fl/fl*^ mice exposed to 18 Gy AIR (Fig. 4b) or WT mice exposed to 18.5 Gy AIR (Fig. 5a) from radiation lethality as 80–100% of animals were dead within 10–12 days of radiation exposure compared with the significantly enhanced survival of mice treated with WT CM (*P*<0.002 Log-rank (Mantel–Cox) test; Fig. 4b and Fig. 5a).

We also purified the EVs from WT/*Porcn*-null BMMΦ CM using conventional ultra-centrifugation methods. EV positive for TSG101, ALIX and CD9 (Fig. 5b) demonstrated WNT activity only when purified from WT BMMΦ CM but not from *Porcn*-null BMMΦ CM (Fig. 5c). Furthermore, the WNT activity (Fig. 5c) and radioprotective activity (Fig. 5a) was entirely removed from the EV-depleted CM effluent. Using an enzyme-linked immunosorbant assay (ELISA) specific for individual WNTs the presence of WNT5a, WNT6 and WNT9a were detected in EVs purified from WT BMMΦ CM (Fig. 5d–f). These data, using two methods of EV purification, indicate that functional WNT ligands are secreted into BMMΦ CM as EV-packaged proteins and these are responsible for the radioprotective effect.

### MΦ-derived WNTs activate β-catenin in irradiated crypt

**Figure 6:**
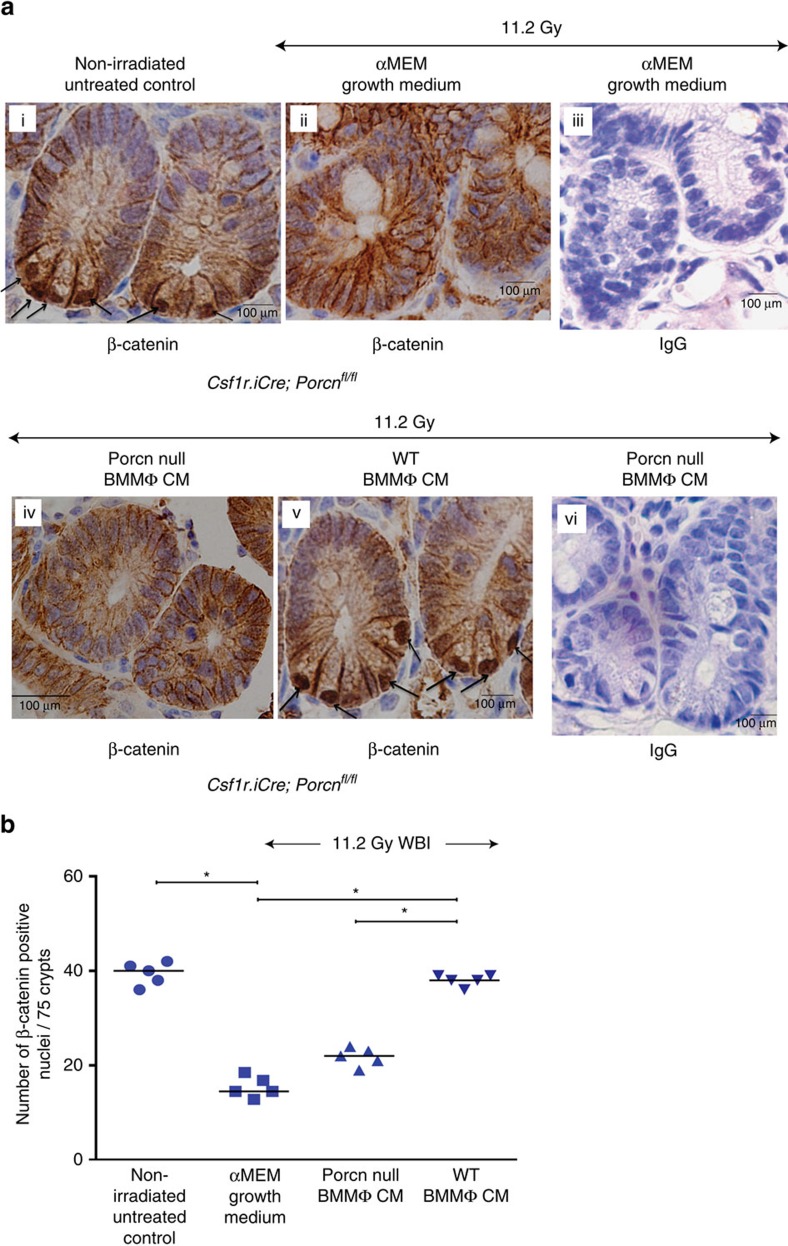
Macrophage-derived WNTs induce β-catenin activity in irradiated crypts. (**a**) Representative microscopic images (× 60 magnification) of jejunal sections immunostained with anti β-catenin antibody to determine β-catenin nuclear localization in *Csf1r.iCre*;*Porcn*^*fl/fl*^ mice. Nucleus stained with haematoxylin. Irradiated *Csf1r.iCre*;*Porcn*^*fl/fl*^ mice receiving WT BMMΦ CM (i.v.) showed more nuclear β-catenin staining (dark brown; indicated with arrows) at the base of the crypt compared with mice receiving *Porcn*-null BMMΦ CM (i.v.) or αMEM growth medium (ii; nucleus stained blue). Fig iii and vi are representative IgG controls indicating lack of staining and thus showing specificity for the anti β-catenin antibody. (**b**) Nuclear β-catenin count: each data point is the average of the number of β-catenin-positive nucleus from 15 crypts per field, 5 fields per mice. Number of β-catenin-positive nucleus in irradiated *Csf1r.iCre*;*Porcn*^*fl/fl*^ mice receiving WT BMMΦ CM is higher compared with *Porcn*-null BMMΦ CM (*P<1E−04 ) or αMEM growth medium (*P<1E−04). Treatment with *Porcn*-null BMMΦ CM and αMEM growth medium following irradiation showed significantly fewer β-catenin-positive nuclei than the non-irradiated control (*P<1.2E−04 and *P<1E−04 respectively; unpaired *t*-test, two-tailed).

Secretory WNTs induce nuclear translocation and activation of β-catenin to drive a gene-expression programme that supports stem cell maintenance, proliferation. Immunohistochemical analysis of jejunal sections from non-irradiated *Csf1r.iCre*;*Porcn*^*fl/fl*^ mice showed characteristic nuclear β-catenin staining with 39±2 of cells being positive for nuclear β-catenin (per 75 crypts; Fig. 6a,b). Similar patterns of nuclear β-catenin staining were also observed in WT mice (Supplementary Fig. 5). Irradiated *Csf1r.iCre*;*Porcn*^*fl/fl*^ mice treated with alpha minimal essential medium (αMEM) or CM form *Porcn*-null BMMΦ had significantly fewer nuclear β-catenin-positive cells (15±2.2 and 22±2, respectively) compared with non-irradiated untreated control (*P*<1E−04 and *P*<1.2E−04, respectively unpaired *t*-test, two-tailed). However, treatment with WT BMMΦ CM significantly increased the number of nuclear β-catenin-positive crypt epithelial cells (38±1.2) compared with αMEM or *Porcn*-null BMMΦ CM-treated cohort (*P*<1E−04 and *P*<1E−04, respectively, unpaired *t*-test, two-tailed; Fig. 6a,b).

Consistent with our immunohistological analysis of nuclear β-catenin expression, the real-time PCR array analysis of β-catenin target genes in crypt epithelial cells showed several-fold increases in mRNA level in irradiated mice treated with WT BMMΦ CM compared with those mice receiving *Porcn*-null BMMΦ CM (Table 1). Altogether these data indicate that BMMΦ-derived WNTs activate the β-catenin pathway in irradiated crypts and that this WNT signal is needed to stimulate proliferation and crypt regeneration.

### BMMΦ WNTs rescue Lgr5^+ve^ ISC from radiation toxicity

**Figure 7:**
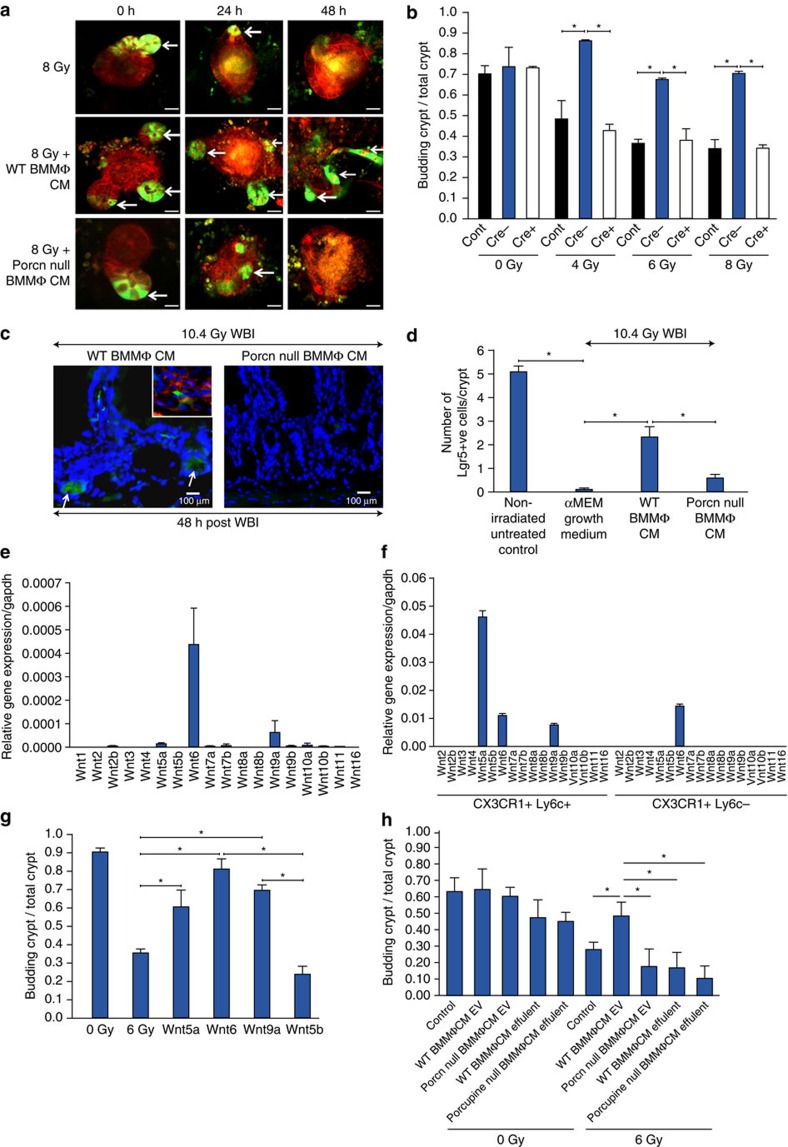
CM from WT BMMΦ rescued the Lgr5^+ve^ ISC population in both *in vivo* and *in vitro*. (**a**) Two-photon microscopic images of organoids from *Lgr5/GFP-IRES-Cre-ERT2* knock-in mouse irradiated and then treated with CM. CM from WT BMMΦ (Cre-) rescued the LGR5^+ve^ (GFP^+ve^ cells) ISC population from radiation injury as indicated by arrow. GFP^+ve^ cells disappeared in organoids treated with/without *Porcn*-null BMMΦ CM (Cre+) within 48 h of exposure to 8 Gy irradiation. Scale bar=50 μM. (**b**) Histograms demonstrating the effect of Cre±CM treatment on crypt organoid growth following irradiation. Treatment groups: 4 Gy versus 4 Gy+Cre−CM (*P<0.013), 6 Gy versus 6 Gy+Cre−CM (*P<0.001), 8 Gy versus 8 Gy+Cre−CM (*P<0.004; unpaired *t*-test, two-tailed). (**c**) Representative images of jejunal sections demonstrating the presence of GFP^+ve^ Lgr5^+ve^ ISCs (arrow) in *Lgr5/GFP-IRES-Cre-ERT2* knock-in mice receiving WT BMMΦ CM. Note, the absence of GFP^+ve^ cells in mice receiving *Porcn*-null BMMΦ CM. Nuclei are stained with DAPI. Top panel Inset: phalloidin (red) staining to show localization of cell membrane. (**d**) Histograms demonstrating the number of GFP^+ve^Lgr5^+ve^ ISCs/crypt in jejunal sections from *Lgr5/GFP-IRES-Cre-ERT2* knock-in mice exposed to irradiation and then treated with *Porcn*-null or WT BMMΦ CM. Irradiated mice receiving WT BMMΦ CM showed higher numbers of GFP^+ve^ cells compared with mice receiving *Porcn*-null BMMΦ CM (*P<0.0002) or αMEM (*P<1.74858E−06; unpaired *t*-test, two-tailed). (**e**) qPCR analysis of RNA from WT BMMΦ demonstrated mRNA expression of *Wnt5a*, *Wnt6* and *Wnt9a*. (**f**) qPCR analysis of intestinal macrophages RNA from WT mice. (**g**) Organoids were exposed to irradiation (6 Gy) and treated with *Porcn*-null BMMΦ CM supplemented with Wnt5a, Wnt6, Wnt9a and Wnt5b (1 μg ml^−1^). Organoid survival was improved with treatment of canonical WNT ligands WNT5a (*P<0.009), WNT6 (*P<0.0001) and WNT9a (*P<8.99662E−05) compared with irradiated control. However, treatment with non-canonical WNT5b failed to rescue organoids from radiation lethality (WNT6 versus WNT5b *P<0.0001; WNT9a versus WNT5b *P<0.0001; unpaired *t*-test, two-tailed). (**h**) Histogram demonstrating the effect of EVs from WT/*Porcn*-null BMMΦ CM on crypt organoid growth following irradiation. Treatment groups: 6 Gy (control) versus 6 Gy+WT BMMΦ CM EV (*P<0.009), 6 Gy+ WT BMMΦ CM EV versus 6 Gy+*Porcn*-null BMMΦ CM EV (*P<0.004), 6 Gy+WT BMMΦ CM EV versus 6 Gy+WT BMMΦ CM effluent (*P<0.0002), 6 Gy+WT BMMΦ CM EV versus *Porcn*-null BMMΦ CM effluent (*P<0.0001; unpaired *t*-test, two-tailed).

To analyse further the specific roles of macrophage-derived WNTs we turned to an *in vitro* primary intestinal organoid culture system exposed to graded doses of radiation. Intestinal crypts were isolated from the *Lgr5/GFP-IRES-Cre-ERT2* knock-in mice to allow visualization of the ISCs and these were cultured *in vitro* as organoids. Irradiation of these intestinal organoids with 2–6 Gy resulted in the loss of budding crypt in a dose-dependent manner (Supplementary Fig. 6). At a radiation dose level of 8 Gy, most of the Lgr5^+ve^ ISCs had disappeared within 48 h post irradiation (Fig. 7a, top panel), resulting in a significant loss of budding crypts with changes in existing crypt morphology indicating inhibition of ISC growth and differentiation in response to radiation exposure.

We next examined the effect of macrophage CM on these crypt organoids exposed to 4–8 Gy irradiation. At every dose of radiation CM from WT BMMΦ rescued the organoids from radiation toxicity and restored the number of budding crypts to untreated levels as represented by ratio of number of budding crypt/total crypt (Fig. 7a,b). In contrast *Csf1r.iCre*;*Porcn*^*fl/fl*^ CM BMMΦ failed to rescue organoids from radiation damage (Fig. 7a,b). A representative example of the effects of WT BMMΦ CM on budding crypt having Lgr5^+ve^ ISCs (GFP^+ve^ indicated with arrow) is shown in Fig. 7a. In these microscopic observations over many experiments and analysis of crypts we observed that CM from WT BMMΦ CM but not *Porcn*-null BMMΦ CM rescued Lgr5^+ve^ ISCs from 8 Gy radiation toxicity. Thus in the case of *Porcn*-null BMMΦ CM most and usually all of the Lgr5^+ve^GFP^+ve^ cells at the base of the budding crypt had disappeared within 48 h of radiation exposure (Fig. 7a).

To study this effect *in vivo* we examined the role of macrophage-derived WNT on ISC survival by exposing *Lgr5/GFP-IRES-Cre-ERT2* knock-in mice to 10.4 Gy WBI and then treated with CM (i.v.) from WT BMMΦ or *Porcn*-null BMMΦ at 1 h and 24 h post WBI. LGR5^+ve^ ISCs had disappeared from the crypt base within 48 h of radiation in mice receiving either αMEM growth medium or CM from Porcn-null BMMΦ (Fig. 7c,d). However, mice receiving WT BMMΦ CM showed significant preservation of LGR5^+ve^ ISCs beyond 48 h post WBI (Fig. 7c,d; *P*<0.0002 compared with mice receiving CM from *Porcn*-null BMMΦ and *P*<1.75E−06 compared with irradiated control receiving αMEM growth medium, respectively, unpaired *t*-test, two-tailed). These results clearly indicate that macrophage-derived WNTs are critical for self-renewal and proliferation of ISCs following radiation *in vivo* and *in vitro*, and that inhibition of macrophage-derived Wnt secretion via deletion of *Porcn* impaired the regenerative response of ISC following radiation toxicity.

### Identification of MΦ WNTs protecting ISC against irradiation

To identify the WNTs responsible for crypt regeneration we first determined those expressed by BMMΦ and intestinal macrophages then used an add-back system to the *Porcn*-null BMMΦ CM that has no WNTs in the organoid cultures to determine their function. RNA was isolated from BMMΦ or flow-sorted intestinal macrophages and subjected to qRT-PCR analysis. This analysis indicated that *Wnt5a*, *Wnt6* and *Wnt9a* were the only WNTs expressed in BMMΦ (Fig. 7e). WNT5a, WNT6 and WNT9a were also predominantly expressed *in* CD11b^+ve^F480^+ve^CX3CR1^+ve^Ly6C^+ve^ intestinal macrophages and WNT6 in CD11b^+ve^F480^+ve^CX3CR1^+ve^Ly6C^−ve^ intestinal macrophages (Fig. 7f). To determine whether these WNT ligands can mediate ISC regeneration following radiation, we examined their effects on the growth of intestinal organoids. Primary crypt cultures were first incubated with CM derived from *Porcn*-null BMMΦ following exposure to 6 Gy of irradiation and then treated with WNT ligands. At 48 h post irradiation, the number of budding crypts was counted and the ratio of budding crypts to total number of crypts determined. Treatment with all the expressed WNTs 5a, 6 and 9a showed significant improvement in organoid growth (*P*<0.009, *P*<0.0001, *P*<8.99662E−05 respectively, unpaired *t*-test, two-tailed) compared with the irradiated control with WNT6 being the most efficient (Fig. 7g). Similar responses were noted when irradiated organoids were treated with WNT in absence of CM indicating that observed organoid growth is a direct effect of WNT supplement (Supplementary Fig. 7a). We have also examined whether the presence or absence of RSPO1 a WNT agonist in BMMΦ CM is contributing to the observed effect of BMMΦ derived WNT in organoids as this has been reported to be involved in crypt repair^9^. RSPO1 was detected in both WT and Porcn-null BMMΦ CM even though the latter cannot rescue crypt development after radiation (Supplementary Fig. 7b) indicating that the observed effect of BMMΦ CM in the organoid cultures is primarily due to presence of WNTs. WNT6 and WNT9a are canonical signalling molecules, while WNT5A can activate both canonical and non-canonical pathways depending on the presence of different receptors^40^,^41^. However, the crypt organoid study indicates that canonical WNT signalling plays a major role in organoid growth against radiation as non-canonical WNT5b failed to induce crypt regeneration (Fig. 7g). In the experiments described above, we also observed that these WNTs were packaged in EVs (Fig. 5d–f). Therefore to confirm the radioprotective effect of these EVs, irradiated organoids were treated with EVs purified from WT or *Porcn*-null BMMΦ CM. Treatment with WT BMMΦ CM-derived EVs rescued organoids from radiation toxicity and improved organoid growth compared with irradiated control. However, treatment with *Porcn*-null BMMΦ CM-derived EV did not show any radioprotective activity and failed to rescue organid growth (Fig. 7h). These data indicate that the canonical WNTs 5a, 6 and 9a are expressed by BMMΦ in EVs and are effective in restoring crypt growth with the highest expressed Wnt 6 being the most efficacious.

## Discussion

Rapid turnover of ISCs makes the intestinal mucosa especially vulnerable to high radiation exposure during radiotherapy. Therefore the gastrointestinal system is an early response organ to radiation. Consequently restoration of intestinal homoeostasis is critical to combat against RIGS. The present study indicates that macrophage-derived WNTs packaged in EVs are required for ISC self-renewal, proliferation and intestinal homoeostasis in response to radiation injury. To establish this involvement of macrophage-derived WNT in repair and regeneration of the intestine we used a mouse model where the gene expressing *Porcn* is selectively deleted in macrophages resulting in the loss of release of WNT ligands. This deletion of *Porcn* in macrophages radio-sensitizes the mice to radiation doses that result in RIGS. Thus these mice die within 10–12 days post exposure and exhibit a disrupted crypt villus architecture compared with WT littermates. In addition we show that macrophage CM containing WNTs but not CM lacking WNTs can rescue the RIGS and result in survival of mice lacking *Porcn* in their macrophages. This rescue, however, is ablated if EVs are removed from the CM. These data can be replicated in intestinal organoid cultures designed to examine the role of LGR5^+ve^ ISCs in stem cell regeneration. This enabled us to identify canonical WNTs 5a, 6 and 9a that are expressed by macrophages able to induce regeneration. These data are consistent with the effects of BMMΦ CM in inducing β-catenin nuclear localization in cryptal epithelial cells *in vivo*. Lethal irradiation of humans results in death due to RIGS and this pathology also limits radiotherapy for abdominal and pelvic cancers. Identification here that macrophage-derived EV-packaged WNTs can rescue this pathology with resultant survival of mice suggests that this might be an effective therapy for mitigation of RIGS following nuclear accidents as well as to increase the efficacy of radiotherapy.

Crypts present within the mouse small intestine have two types of stem cells. Bmi1 positive ISCs that are long-lived, label-retaining stem cells present at the +4 position of the crypt base. These Bmi1^+ve^ ISCs interconvert with more rapidly proliferating LRG5^+ve^ stem cells known as CBCs that express markers including *Lgr5*, *Olfm4*, *Lrig1* and *Ascl2*.

These CBCs are also active stem cells, in as much as they are primarily involved in self-renewal and differentiation^13^,^42^,^43^,^44^,^45^,^46^,^47^,^48^,^49^. Although several reports suggested that intestinal stroma is the major source of WNT in the intestine^17^,^50^ the involvement of stromal WNTs in intestinal homoeostasis is not clear. Different cell types of intestinal stroma including endothelial cells, macrophages, neurons, fibroblasts and myofibroblasts could produce a cocktail of redundant WNT ligands maintaining intestinal homoeostasis *in vivo*. However, inhibition of WNT release from intestinal myofibroblast^50^ or from intestinal epithelium^1^ does not affect intestinal homoeostasis. Pharmacological depletion of PORCN affecting both stromal and epithelial WNT release has shown that WNTs are required for recovery from radiation while depletion of WNT secretion by ablation of *Porc* using the *Villin-cre* in the intestinal epithelium indicated that this epithelium was not the WNT source during recovery from radiation. The present study has demonstrated that macrophages are the critical source of WNT for ISC regeneration as BMMΦ CM deficient in WNT obtained from the *Csf1r.iCre*;*Porcn*^*fl/fl*^ mice failed to rescue Lgr5^+ve^ ISCs following radiation injury. Moreover, macrophage-derived WNTs were secreted and trafficked through EVs as EV-depleted BMMΦ CM is unable to mitigate radiation toxicity. However, macrophage-derived WNTs are not required for maintenance of normal intestinal homoeostasis as there were no phenotypic changes observed in the crypt architecture between non-irradiated WT and *Csf1r.iCre*;*Porcn*^*fl/fl*^ mice.

Interestingly, in other organs macrophage WNTs are also required following injury. For example, macrophage WNT7b is required for kidney epithelial repair^28^. In addition, macrophage WNT signalling is required for stem cell regeneration in liver following damage^29^. Thus there appears to be a developing paradigm that macrophages deliver these important developmental molecules in a spatial and temporal specific way in adult animals to re-capitulate developmental processes to effect repair. In our studies on radiation repair we showed these macrophage-synthesized WNTs were contained within the EV fraction. While it is possible that the WNTs are in a contaminating vesicular fraction of another type it is also creditable that they are packaged in exosomes. While this remains to be formally proven it maybe that macrophage-derived EV containing WNTs are responsible in all these examples indicating a paradigm for macrophage-regulated tissue repair. However, in some cases this delivery of WNTs appears to enhance pathology for example in cancer where macrophages may detect tissue damage caused by tumour growth^51^ and respond by WNT production such as WNT7b. This macrophage-derived Wnt7b has been shown to enhance the growth and metastasis of tumours^52^. Understanding the control and molecular basis of these WNT signalling pathways in adults might therefore be of therapeutic use both to enhance repair as in this case against radiation-induced injury or to inhibit growth as in cancer.

## Methods

### Animals

Porcupine floxed (*Porcn*) embryos (FVB female donor+ICR male donor) were kindly provided by Dr J. Rossant (Hospital for Sick Children Research Institute, Toronto, Canada)^21^. Embryos were transferred into oviducts of FvB females and the resultant pups bred to homozygosity after first back-crossing to the transgenic cre-recombinase strain, FVB.*Csf1r-icre* males for 3 generations (N3) to generate *Tg(Csf1r.iCre)Jwp.-Porcn*^*tmros*^) mice. Thereafter mice were inter-bred and cre-deleted mice compared with littermate cre-negative controls referred to as WT. All genotyping of mice was performed by PCR using the following primers (5′-3′): PorcnRecF1: 5′-CTGTTAAACCAAGACATGACCTTCA-3′; PorcnRecR1, 5′-TAACTAGGACGCTTTGGGATAGGAT-3′; and PorcnRecR3: 5′-GTTCTGCCTTCCTAACCCATATAAC-3′ (ref. 21). Amplicon sizes for primer combination F1-R1 representing floxed allele is 248 bp and primer combination F1-R3 representing the Porcn-deleted allele is 386 bp. For Cre-recombinase forward: 5′-CTCTGACAGATGCCAGGACA-3′; and Cre reverse: 5′-TCTCTGCCCAGAGTCATCCT-3′. For QPCR of genomic DNA and mRNA: Porcn-qPCR-F 5′-GCTGTCTCCTGCCTACTGTCCA-3′ Porcn-qPCR-R 5′-TGCTTGCATGCTTCAGGTAAGA-3′. All procedures involving mice were performed in accordance with National Institutes of Health regulations concerning the care and use of experimental animals. Experimental procedures were approved by Institutional Animal Care and Use Committee of the Albert Einstein College of Medicine and University of Kansas Medical Center. Female mice (6–8 weeks old) for both *Csf1r.iCre*;*Porcn*^*fl/fl*^ and WT genotype were used for all the experiments. Male (6–8 weeks old) *Lgr5/GFP-IRES-Cre-ERT2* knock-in mouse were used for all the experiments.

### Isolation of BMMφ

Bone marrow was isolated from both femurs and tibias of adult females *Porcn*;*Csf1r.Cre* mice and their WT littermate controls with >95% purity^53^. Tibias were flushed and bone marrow cells were seeded into 10 cm^2^ tissue culture plates and cultured in alpha MEM (Cellgro) containing 10% (v/v) fetal bovine serum (FBS) and 1% penicillin–streptomycin, supplemented with M-CSF 10^4^ U ml^−1^ for 24 h. To generate fully differentiated monocyte-derived macrophages non-adherent cells were transferred to petri dishes and cultured for 6–7 days at 37 °C. Macrophage CM was collected and then concentrated (10-fold) with centrifugal filter units (Millipore, Billerica, MA) before use for *in vivo* treatment (500 μl per mice, i.v.). For *in vitro* organoid studies mature bone marrow macrophages were cultured for 48 h in serum deprived medium (0.5% v/v FBS) and then CM was concentrated (10-fold) with centrifugal filter units. For pharmacological inhibition of Porcupine WT BMMφ were treated with C59, Porcupine Inhibitor II (Calbiochem; 20 μM). After 2 h of incubation cell culture medium (complete alpha MEM) having C59 was withdrawn and replaced with new medium without C59. One hour after WT BMMφ CM was collected and concentrated for TOPFLASH assay (below).

### TCF/LEF (TOPFLASH) reporter assay

To determine the canonical WNT activity in BMMφ CM HEK293 cells (Signosis, Santa Clara, CA) having TCF/LEF luciferase reporter construct were treated with WT BMMφ CM or Porcn-null BMMφ CM or EV-depleted WT BMMφ CM or C59-treated WT BMMφ CM. LiCl (10 mM) treatment was used as positive control for luciferase activity. Luciferase activity was determined 24 h after using Dual-Luciferase Reporter Assay System (Promega) as per manufacturer's protocol. HEK293 cells having FOPFLASH construct (mutated TCF/LEF-binding site) were used as negative control. WNT activity was consistent between different batches of CM prepared under the same conditions.

HEK293 (human embryonic kidney) cell line was routinely characterized in the lab based on morphology and gene-expression patterns. Cells were confirmed to be free of mycoplasma contamination.

### EV purification from WT BMMφ CM using EV Isolation Reagent

EV depletion in WT BMMφ CM was performed using Total Exosme Isolation Reagent (from cell culture media; Invitrogen) as per manufacturer's protocol. Effluent following EV purification was considered as depleted/EV-free fraction of WT BMMφ CM. Depletion of EV was confirmed by immunoblotting with exosome marker TSG101 (1:250; #AB125011, Abcam, Cambridge, MA), ALIX (1:250; #AB117600, Abcam) and CD9 (#AB92726, Abcam). Absence of positive bands for all three exosomal markers in depleted fraction confirmed the EV depletion. However, positive bands for TSG101, ALIX and CD9 were detected in WT BMMφ CM and the purified EV fraction (All un-cropped western blots can be found in Supplementary Fig. 8). Average yield of EV was 60 μg per 50 ml of BMMφ CM. EV at 100 μg ml^−1^ was used for *in vitro* assays.

### Purification of EV from BMMφ CM by conventional method

EVs were isolated from the WT/Porcn-null BMMφ CM as previously described^54^. Briefly, BMMΦ CM were centrifuged at 2,000*g* for 20 min and 16,500*g* for 30 min to remove other types of vesicles, such as microvesicles. The resulting cell-free medium was subjected to ultra-centrifugation at 100,000*g* for 1 h to generate an EV pellet that was washed once with phosphate-buffered saline (PBS). The amount of EV protein recovered was assessed using detergent-compatible protein assay (Bio-Rad) according to the manufacturer's instructions. Average yield of EV was 75 μg per 50 ml of BMMφ CM. Purified EV at the concentration of 100 μg ml^−1^ and 200 μg ml^−1^ was used for TOPFLASH assay and *ex vivo* organoid assay, respectively.

### ELISA of EV-packaged WNT

EV-packaged WNT was detected using ELISA starter accessory Kit (E101, Bethyl Laboratories, Montgomery, Texas) according to manufacturer's protocol. Biotin-conjugated antibodies against WNT5A, WNT9A (Bioss antibodies, Woburn, MA) and WNT6 (Novus Biologicals, Littleton, CO) was used for the ELISA. Purified EV with the concentration of 100 μg ml^−1^ was used for each assay.

### Irradiation Procedure

WBI was performed on anaesthetised mice (intraperitoneal ketamine and xylazine 7:1 mg ml^−1^ for 100 μl per mouse) using a Mark I-68 A Cs-137 irradiator (JL Shepherd and Associates, San Fernando, CA) at a dose rate of 236 cGy min^−1^ following biosafety guidelines of Albert Einstein College of Medicine.

AIR was performed on anaesthetised mice (with a continuous flow 1.5 l min^−1^ of 1.5% isoflurane in pure oxygen) using the small animal radiation research platform (SARRP, XStrahl, Surrey, UK). A 2 cm area of the mice containing the GI was irradiated (Fig. 2), thus shielding the upper thorax, head and neck as well as lower and upper extremities, protecting a significant portion of the bone marrow, thus inducing predominantly RIGS. A radiation dose of 18 Gy was delivered to the midline of the GI, ensuring homogeneous delivery by performing half of the total irradiation from the anterior-posterior direction and the second half from the posterior–anterior direction. The total irradiation time to deliver the intended dose was calculated with respect to dose rate, radiation field size and fractional depth dose to ensure accurate radiation dosimetry.

### Histology

Since radiation doses >8 Gy induces cell cycle arrest and apoptosis of the crypt epithelial cells within day 1 post-radiation, resulting in a decrease in regenerating crypt colonies by day 3.5 and ultimately villi denudation by day 7 post-radiation exposure^2^, we killed animals when moribund or at 3.5 and 7 days after WBI or AIR for time course experiments and intestines were collected for histology. The intestine of each animal was dissected, washed in PBS to remove intestinal contents and the jejunum was fixed in 10% neutral-buffered formalin before paraffin embedding. Tissue was routinely processed and cut into 5 μm sections for haematoxylin and eosin and immunohistochemical staining. All haemotoxylin and eosin (HE) (Fisher Scientific, Pittsburgh, PA) staining was performed at the Histology and Comparative Pathology Facility in the Albert Einstein Cancer Center.

### Crypt Proliferation Rate

To visualize villous cell proliferation, each mouse was injected intraperitoneally with 120 mg kg^−1^ BrdU (Sigma-Aldrich, USA) 2 to 4 h before killing and mid-jejunum was collected for paraffin embedding and BrdU immunohistochemistry. Tissue sections were routinely deparaffinized and rehydrated through graded alcohols and incubated overnight at room temperature with a biotinylated monoclonal BrdU antibody (Zymed, South Francisco, CA). Nuclear staining was visualized using Streptavidin-peroxidase and diaminobenzidine (DAB) and samples were lightly counterstained with haematoxylin. Jejunum from mice, not injected with BrdU, was used as a negative control. Murine crypts were identified histologically according to the criteria established by Potten *et al*.^55^. Digital photographs of crypts were taken at high (× 40–60) magnification (Zeiss AxioHOME microscope) and crypt epithelial cells in intestinal sections were examined using ImageJ software and classified as BrdU positive if they grossly demonstrated brown-stained nuclei from DAB staining or as BrdU negative if they were blue stained nuclei. The proliferation rate was calculated as the percentage of BrdU-positive cells over the total number of cells in each crypt. A total of 60 crypts were examined per animal.

### Determination of Villi Length and Crypt Depth

Crypt depth was independently and objectively analysed and quantitated in a blind manner from coded digital photographs of crypts from HE-stained slides using ImageJ 1.37 software to measure the height in pixels from the bottom of the crypt to the crypt villus junction. Villi length was determined by measuring the length from the crypt villus junction to villous tip. This measurement in pixels was converted to length (in μm) by dividing with the following a conversion factor (1.46 pixels μm^−1^).

### β-catenin immunohistochemistry of mouse jejunum

β-Catenin immunohistochemistry was performed in paraffin-embedded sections of mouse jejunum^56^. Before immunostaining antigen retrieval was performed by heating slides in pH 6.0 citrate buffer at 100 °C for 20 min in a microwave oven at 500 W using antigen retrieval solution (10 mM Tris and 1 mM EDTA, pH 9.0). Non-specific antibody binding was blocked for 20 min by incubation with 0.05% w/v BSA in PBS. Tissue was stained using the anti-β-catenin Antibody (1:100 dilution; BD Transduction Laboratories, Franklin Lakes, NJ; #610154) at room temperature for 2 h followed by staining with horseradish peroxidase-conjugated Anti-Mouse Antibody (Dako, Denmark) at room temperature for 1 h. Peroxidase activity was detected by adding DAB substrate. Nucleus was counter-stained with haematoxylin (blue). β-Catenin-positive nucleus (stained dark brown) was calculated from 15 crypts per field, 5 fields per mice.

### Isolation of Intestinal Epithelial Cells

Intestinal epithelial cells were prepared from the jejunum of adult male C57Bl6 mice^57^. Mice were anaesthetized and a catheter was inserted into the intestine through an incision in the most proximal part of duodenum. A second incision was made just proximal to the caecum and the entire small intestine was perfused with ice-cold PBS and then flushed twice with ice-cold PBS plus 1 mM dithiothreitol (DTT). The duodenum and ileum were discarded and the entire jejunum was tied at the distal end and filled to distension with isolation citrate buffer (0.9% w/v NaCl, 1.5 mM KCl, 27.0 mM Na Citrate, 8.0 mM KH2PO4 and 5.6 mM Na2HPO4, pH 7.3) heated to 37 °C for 15 mins. After incubation, the jejunum was emptied and filled with 5 ml ethylene diamine tetra acetic acid (EDTA) buffer (0.9% w/v NaCl, 8 mM KH2PO4, 5.6 mM Na2HPO4, 1.5 mM Na2-EDTA, pH 7.6, plus 0.5 mM DTT and 0.23 mM PMSF; Sigma-Aldrich, St Louis, MO). Each jejunum was then physically manipulated and tapped allowing the cells to separate from the interior surface. The jejunum was finally rinsed twice with 5 ml of EDTA buffer and all the fluid containing epithelial cells was collected, centrifuged at 300*g* for 5 min, washed twice with 20 ml of balanced salt solution (BSS) containing 135 mM NaCl, 4.5 mM KCl, 5.6 mM glucose, 0.5 mM MgCl2, 10 mM HEPES and 1.0 mM CaCl2, pH 7.4, and the cells suspended in 2 ml of the same solution. Cell numbers were determined with haemocytometer and viability (>90±5%) was assessed using trypan blue exclusion.

### Isolation and flowcytometric sorting of intestinal MΦ

Isolation of Intestinal lamina propria cells was performed by following a method established previously^58^ with slight modifications. Small intestines were washed with three times with HBSS (Ca/Mg-free), and fat and Peyer's patches were removed. Small intestines were then opened longitudinally, cut into 1-cm pieces, and incubated in HBSS containing 5 uM EDTA+5%FBS+1μM DTT. Tissue was then digested 0.14 Wünsch U ml^−1^ Liberase (Sigma) for 30 mins at 37 °C on a rotor. The digested cell suspension was then passed through 100 μm cell strainers. Isolated intestinal cells were stained with CD45 Percpcy5.5 (0.35 μl per 100 μl; #45–0451–82, eBioscience, San Diego, CA) CX3CR1 PE-TexasRed (1.5 μl per 100 μl; #149013, Biolegend, San Diego, CA) F4/80 AF647 (8 μl per 100 μl; #MCA497A647, Bio-Rad, Hercules, CA), CD11b eflour605 (2 μl per 100 μl; #83–0112–42, eBioscience) Ly6C PE/cy7 (1 μl per 100 μl; #128018, Biolegend) Ly6G APC/cy7 (0.3 μl per 100 μl; #127624, Biolegend) and were subjected to flowcytometric sorting to purify intestinal macrophages using FACS Aria machine (BD).

### Real-time PCR to determine β-catenin target genes mRNA level

To compare the mRNA levels of β-catenin target genes in intestinal crypt cells from irradiated mice treated with *Porcn*-null BMMΦ CM or WT BMMΦ CM real-time PCR were performed using real-time array system from Qiagen. RNA was isolated from crypt cells using RNeasy mini kit from Qiagen. Preparation of cDNA followed by real-time PCR array was performed according to manufacturer protocol.

### FITC-dextran permeability assay

At day 5 post WBI mice were gavaged with 0.6 mg g^−1^ body weight of a FITC-dextran solution (4,000 kD size, Sigma). In all, 4 h after gavage mice were killed and serum was obtained with cardiac puncture^59^. Samples were measured in a 96-well plate using a Flexstation ii 384 multiwell fluorometer (Molecular Devices). A standard curve was constructed using mouse serum having increasing amounts of FITC-dextran to determine the serum levels of FITC-dextran in different treatment groups.

### Preparation *in vitro* culture of intestinal crypt organoids

Small intestine from Lgr5-GFP mice, or their littermates control mice was used for Crypt isolation and development of *ex vivo* organoid culture^60^,^61^,^62^. The tissue was scraped for removing villi and chopped into ∼5 mm pieces. Then tissue was washed with cold PBS, and incubated in 2.5 mM EDTA in PBS for 60 min on ice. The tissue fragments were suspended vigorously with a 10-ml pipette in cold 10% v/v FBS, yielding supernatants enriched in crypts. Crypt fractions were centrifuged at 300*g* for 5 min at 4 °C and diluted with advanced DMEM/F12 (Invitrogen) containing B27, N2, 1 μM n-Acetylcysteine, 10 mM HEPES, penicillin/streptomycin, and Glutamax (all Invitrogen). Samples were passed through 100 μm filters (BD Biosciences), and centrifuged at 275*g* for 5 min at 4 °C and single cells were discarded. Crypts were embedded in extracellular matrix (provided from NCI) and seeded on pre-warmed 24-well plate. After the matrix solidified, advanced DMEM/F12 medium containing 50 ng ml^−1^ EGF (Invitrogen), 100 ng ml^−1^ Noggin (Peprotech), 1 μg ml^−1^ RSPO1 was overlaid. Growth factors were added every other day and the entire medium was changed twice a week. Passage was performed at day 7. The number of organoids per well was counted on microscopic images. The images of organoids were acquired using fluorescent microscopy (Nikon, TE2000-U) and two-photon microscopy (Nikon, A1RMP). For WNT supplementation experiments WNT5A, WNT9A (R&D Systems) and WNT6 (Novus) was used with the concentration of 1 μg ml^−1^. Total number of crypt structures and number of budding crypts were counted and expressed as a ratio of budding crypts/total crypt structure.

### Statistics

Mice survival/mortality in different treatment group was analysed by Kaplan–Meier statistics as a function of radiation dose using Graphpad Prism-6.0 software for Mac. Mice were sorted randomly after genotyping to each experimental and control group. Minimum number of mice used for survival/mortality study was *n*=10 per group. For intestinal sampling regions were chosen at random for digital acquisition for quantitation. Digital image data was evaluated in a blinded manner as to treatment. A two-sided Student's *t*-test was used to determine significant differences between experimental cohorts (*P*<0.05) with representative standard errors of the mean.

### Study approval

All the animals were maintained in the animal maintenance facilities and all animal studies were performed under the guidelines and protocols of the Institutional Animal Care and Use Committee of the Albert Einstein College of Medicine and University of Kansas Medical Center.

### Data availability

The authors declare that all data supporting the findings of this study are available within the article and its Supplementary Information files or from the corresponding author on reasonable request.

## Additional information

**How to cite this article**: Saha, S. *et al*. Macrophage-derived Extracellular Vesicle-packaged WNTs rescue intestinal stem cells and enhance survival after radiation injury. *Nat. Commun.* **7**, 13096 doi: 10.1038/ncomms13096 (2016).

## Supplementary Material

Supplementary Information Supplementary Figures 1-8

## Figures and Tables

**Table 1:** Real-time PCR analysis to determine mRNA levels of different **β**-catenin target genes in crypt epithelial cells.

Wnt target genes	11.2 Gy WBI*Csf1r.iCre*;*Porcn*^*fl/fl*^ mice+Porcn-null BMMΦ CM versus *Csf1r.iCre*;*Porcn*^*fl/fl*^ mice+WT BMMΦ CMLog2 (fold change)
Ascl2	9.60±1.4
Lgr5	4.44±0.9
Plaur	4.05±0.75
Mmp9	3.82±0.82
Ctgf	3.08±1.1
cMyc	2.66±0.62
Ptgs2	2.52±0.86
Twist1	2.49±0.23
Axin2	2.30±0.12
Lef1	2.21±0.08
Fosl1	2.04±0.02
Pou5f1	2.01±0.03
