# Supplementary material for: Macrophage-derived extracellular vesicle-packaged WNTs rescue intestinal stem cells and enhance survival after radiation injury
Source: Nat Commun. 2016 Oct 13;7:13096. doi: 10.1038/ncomms13096 (PMC5065628; doi:10.1038/ncomms13096)
Supplement: Supplementary Information — Supplementary Figures 1-8 [file ncomms13096-s1.pdf]

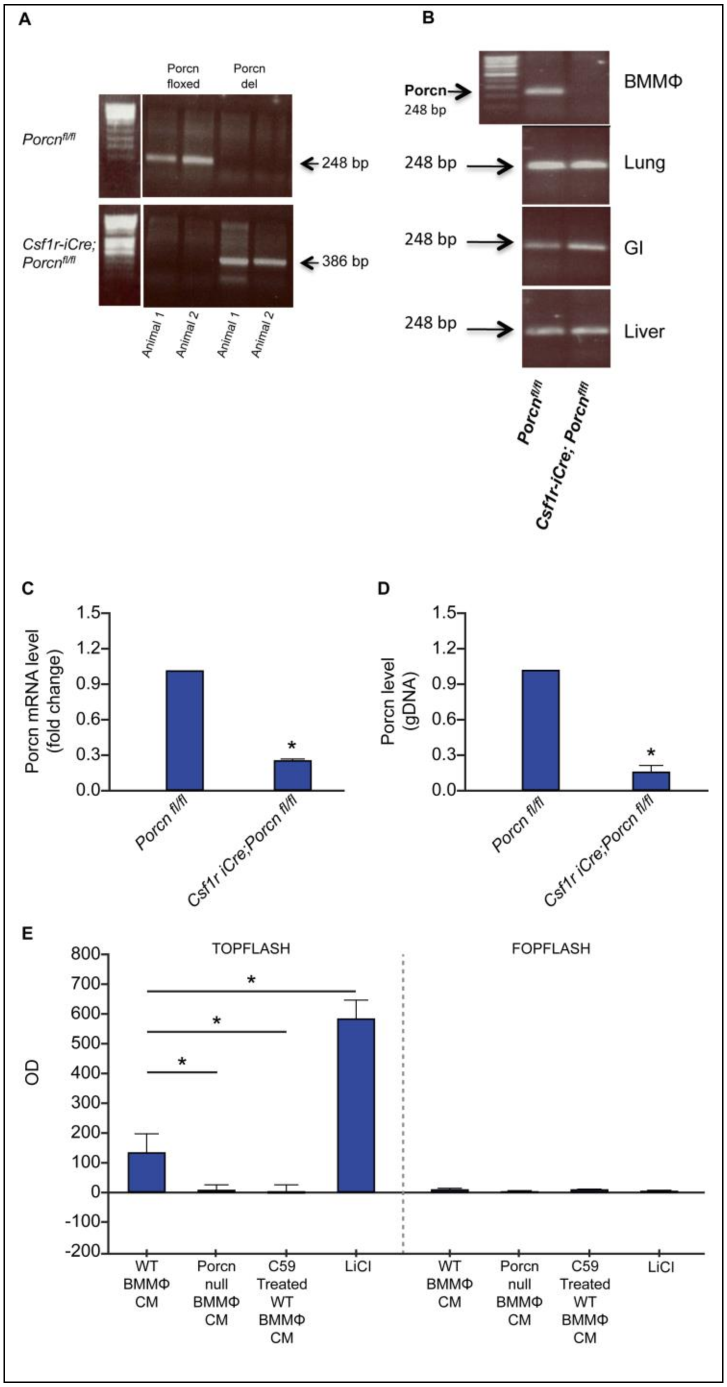

Supplementary Figure 1. Characterization of *Csf1r.iCre;* Porcn$^{fl/fl}$ mice.

A) PR of isolated BMMϕ DNA using primers to detect using specific primer pairs the expression or absence of *Porcn* (floxed, 248bp) sequence and the generation of the recombined allele (del, 386bp) in two different animals. **B)** PCR for the deleted allele of *Porcn* of DNA from BMMϕ, lung, crypt epithelial cells nd liver of *Csf1r.iCre;* Porcn$^{fl/fl}$ and Porcn$^{fl/fl}$ mice. **(C-D)** Real time quantitative PCR analysis to deterine mRNA (**C**) and genomic DNA (D) levels of *Porcn* in isolated BMMϕ from *Csf1r.iCre;Porcn*$^{fl/fl}$ and *Porcn*$^{fl/fl}$ mice. **E)** TCF/LEF reporter assay. HEK293 cells having an integrated TCF/LEF luciferase reportr construct were treated with BMMϕ CM from *Csf1r.iCre;*Porcn$^{fl/fl}$ or WT mice (n=5/group) or C59 (PORCN inhibitor) treated WT BMMϕ CM or with LiCl (positive control). Treatment with WT BMMϕ CM showed higher luciferase activity compared to *Csf1r.iCre; Porcn*$^{fl/fl}$ BMMϕ CM (*, $p<0.006$) or CM from C59 treated WT BMMϕ (*, $p<0.01$) (unpaired *t*-test, two-tailed). HEK293 cells having FOPFLASH construct did not show luciferase activity with different treatments.

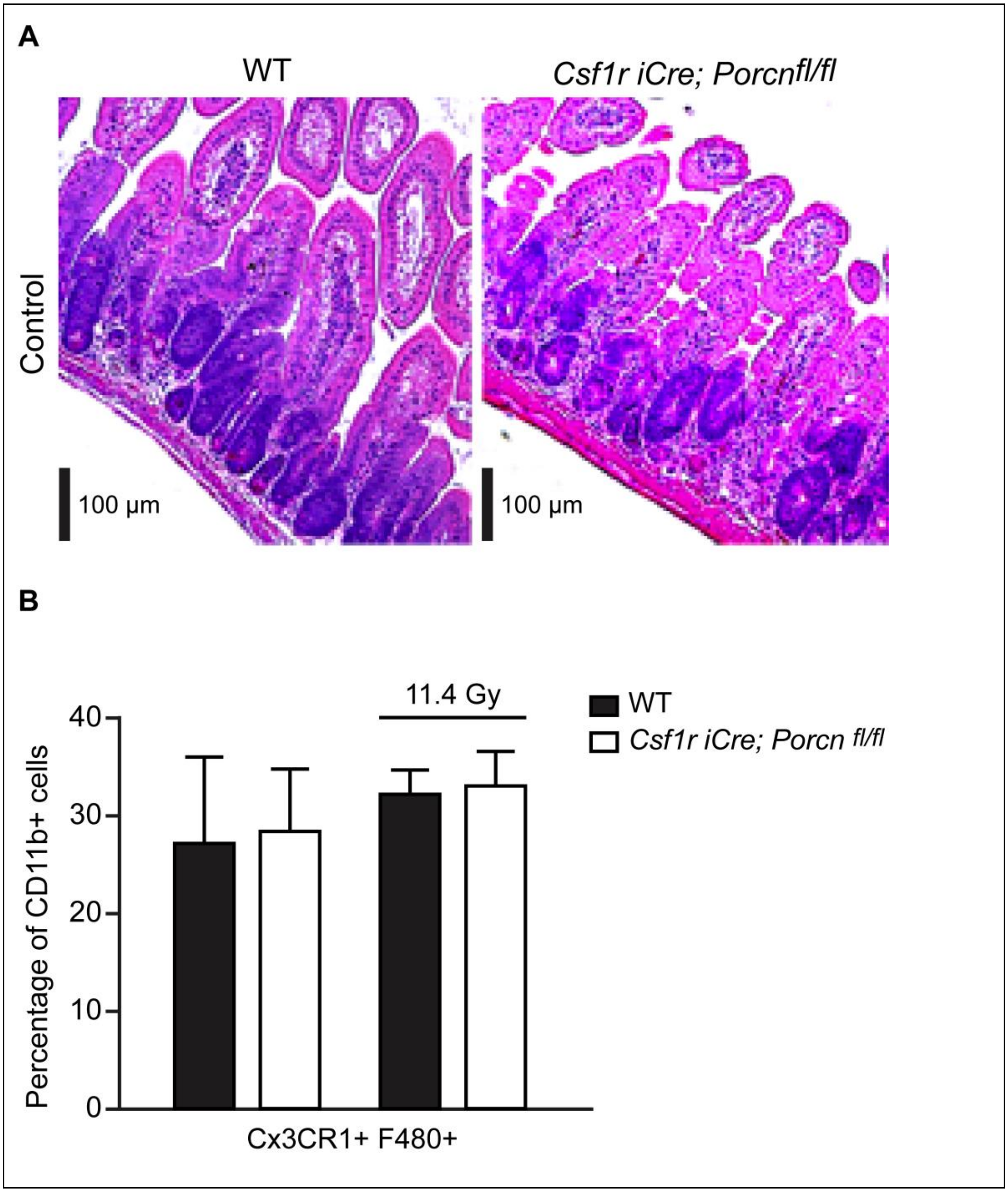

Supplementary Figure 2. *Csf1r.iCre;Porcn$^{fl/fl}$* mice showed normal intestinal morphology, intestinal macrophage count similar to WT mice.

A. H&E staining of jejunum section from *Csf1r.iCre;Porcn$^{fl/fl}$* and WT mice. No significant differences in crypt villus structure were noted in *Csf1r.iCre;Porcn$^{fl/fl}$* mice compared to WT mice.

B. Flow cytometric analysis of intestinal macrophages obtained from *Csf1r.iCre;Porcn$^{fl/fl}$* and WT mice to determine the effect of irradiation on intestinal macrophage population size. There was no significant difference in percent of CD11b+ intestinal macrophages expressing CX3CR1 and F480 (markers specific for intestinal macrophages) in *Csf1r.iCre; Porcn$^{fl/fl}$* and WT mice post irradiation (48hrs post WBI).

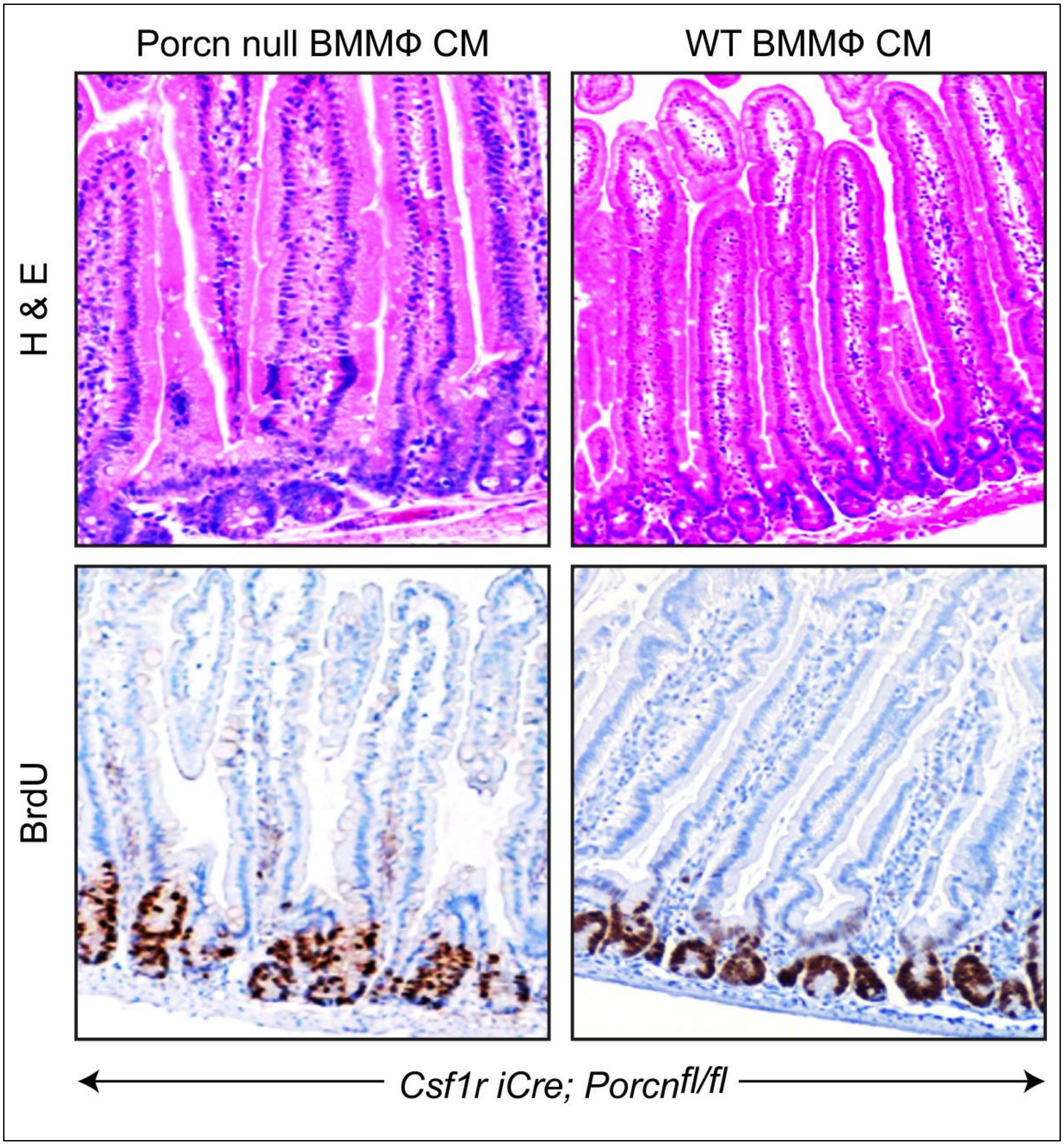

Supplementary Figure 3. CM from *Porcn* null or wild type BMMϕ could not affect crypt villus morphology or cell proliferation in non-irradiated mice *Csf1r.iCre;Porcn$^{fl/fl}$* **mice .**

H&E (Top) and BrdU immunohistochemistry (Bottom) of transverse sections of jejunum from non-irradiated *Csf1r.iCre;Porcn$^{fl/fl}$* mice treated with *Porcn* null or wild type BMMϕ CM. Treatment with these two-conditioned media did not alter crypt villus morphology or cell proliferation.

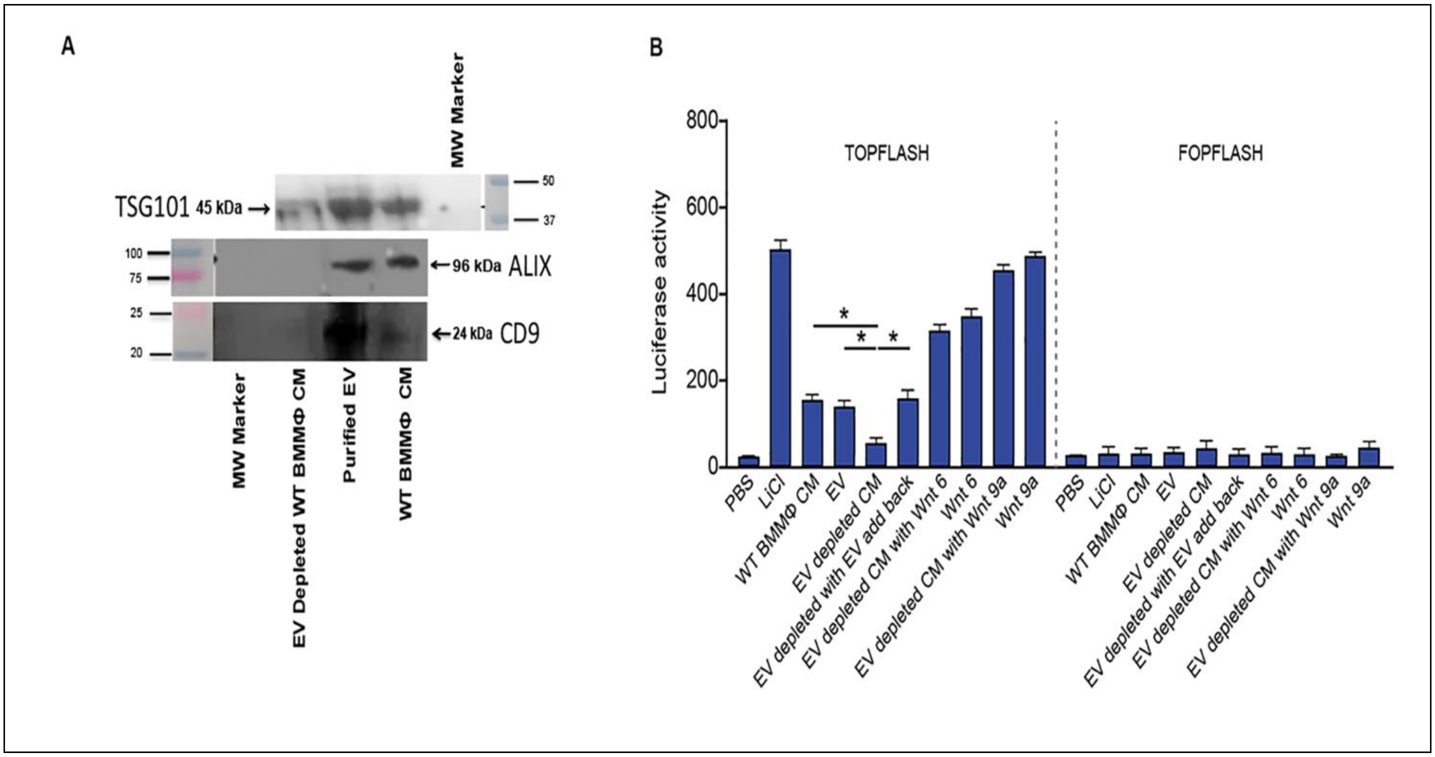

Supplementary Fig 4. Depletion of EV in WT BMMΦ CM reduces canonical WNT activity.

A) Immunoblot to detect EV markers TSG101, ALIX and CD9 in WT BMMΦ CM, EV depleted WT BMMΦ CM and EV purified fraction. Positive bands for all three EV markers were detected in WT BMMΦ CM and EV purified fraction but not in EV depleted WT BMMΦ CM.

B) TCF/LEF reporter assay. HEK293 cells having TCF/LEF luciferase reporter construct were treated with LiCl as a positive control or with WT BMMϕ CM, purified EV (100ug/ml), EV depleted fraction of WT BMMϕ CM, depleted fraction with EV add back as indicated. Treatment with WT BMMϕ CM or purified EV or EV add back to depleted fraction showed higher luciferase activity compared to EV depleted WT BMMϕ CM (*, $p<0.003$; *,$p<0.005$ and *,$p<0.006$ respectively) (unpaired *t*-test, two-tailed). Treatment with EV depleted fraction from WT BMMϕ CM containing recombinant WNT6 or WNT9a (1µg/ml) showed presence of WNT activity in the depleted fraction as observed by the Luciferase activity. No significant difference was observed in Luciferase activity with EV depleted fraction with WNT6 or WNT9a compared to WNT6 or WNT9a alone.

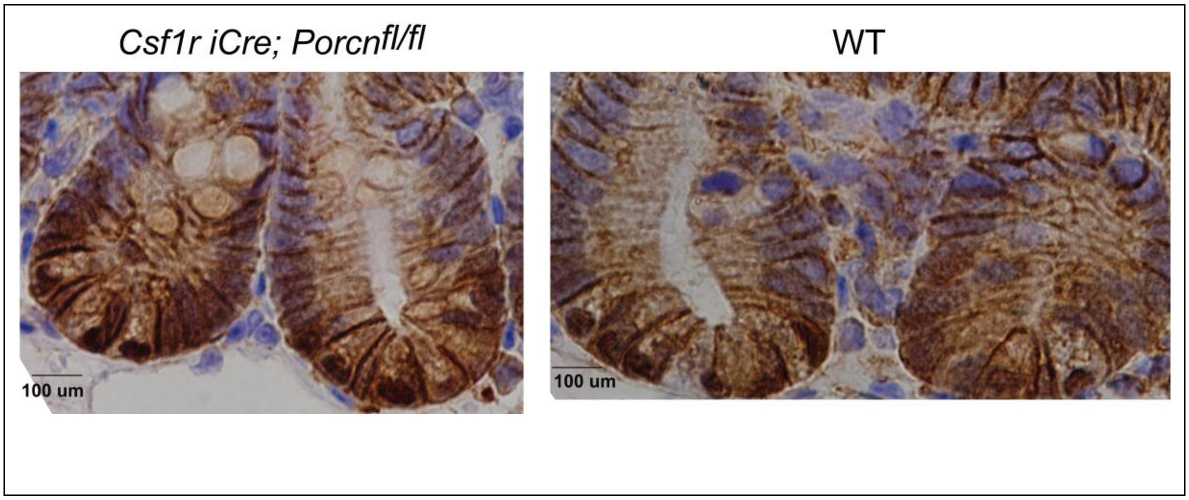

Supplementary Figure 5. Representative microscopic images of jejunal sections immunostained with anti β-catenin antibody to determine β-catenin nuclear localization in WT and *Csf1r.iCre;Porcn*$^{fl/fl}$ mice. Nucleus stained with hematoxylin. No significant differences were observed in nuclear translocation of β-catenin in non irradiated *Csf1r.iCre;Porcn*$^{fl/fl}$ and WT mice.

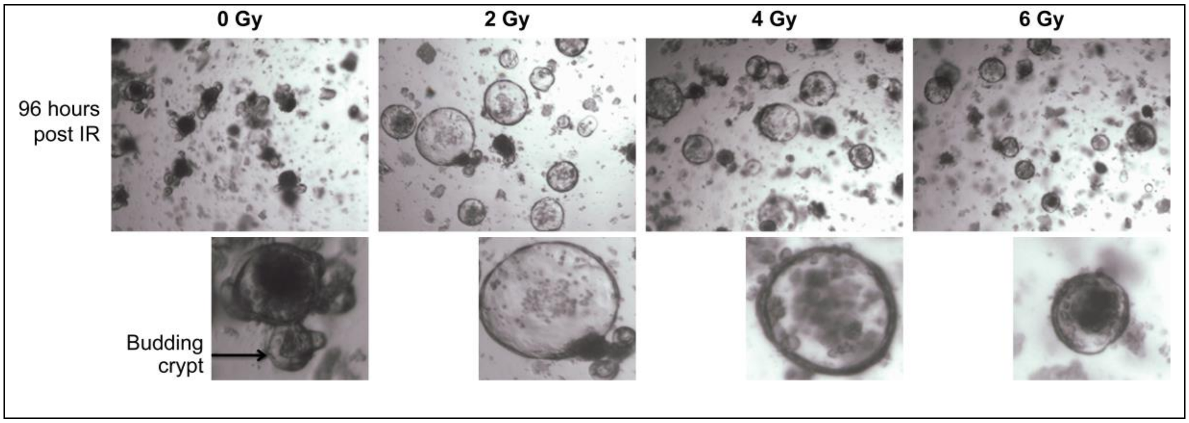

Supplementary Fig 6. Effect of different doses of radiation on crypt organoid structure.

Radiation reduces the crypt organoid structure in a dose dependent manner.
Phase contrast photomicrographs of intestinal organoid cultures to determine the development of crypts. Irradiation of these intestinal organoids (2-6 Gy) resulted in loss of budding crypt (indicated by arrow) in a dose dependent manner. Loss of budding crypt was higher at 6Gy compared to 2Gy at 96hr post irradiation. Note the absence of budding crypts along with significant changes in organoid morphology at the dose level of 4-6 Gy.

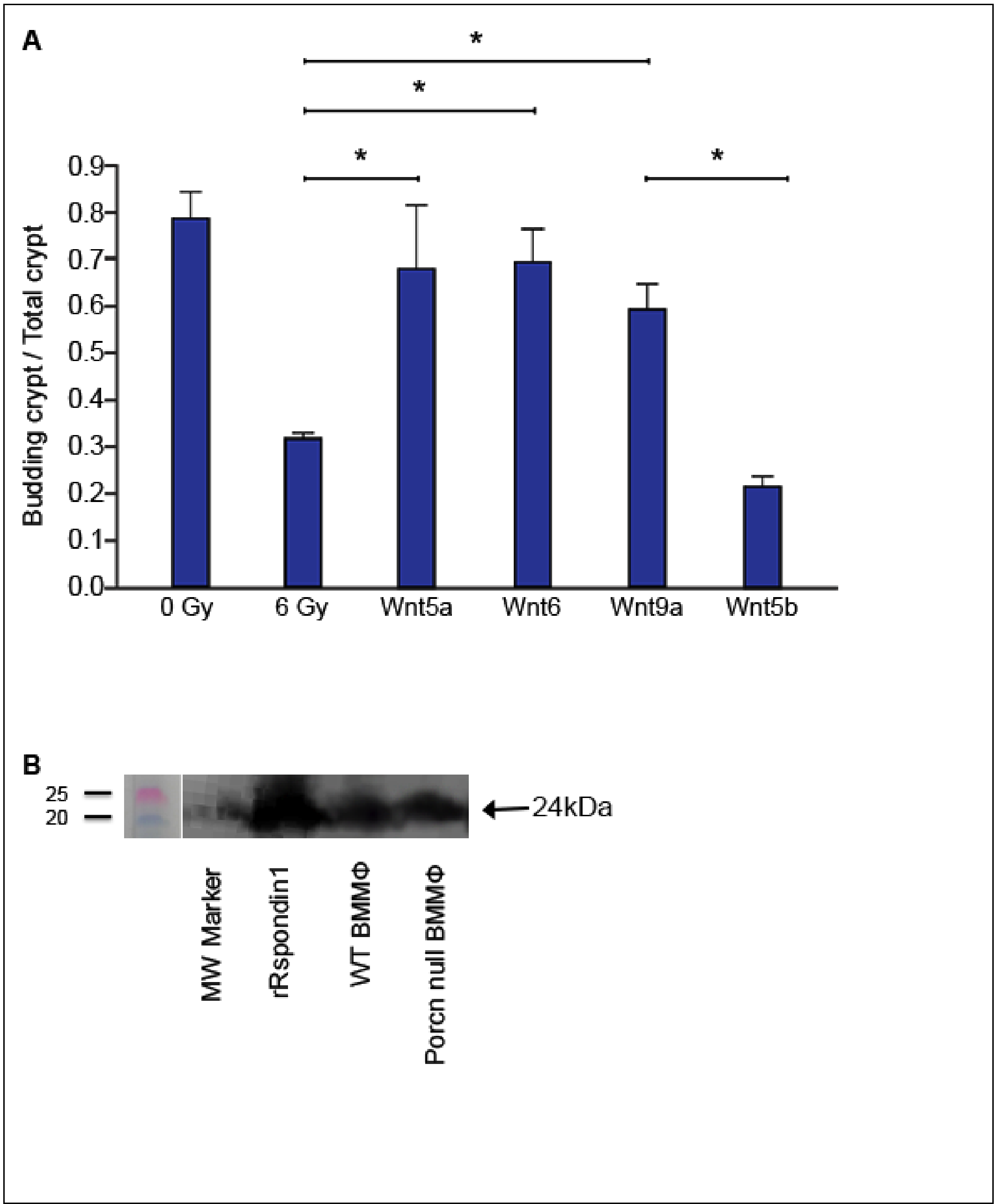

Supplementary Fig 7. A. Determination of the role of exogenous WNTs in organoid growth after radiation exposure. Wnt5a, Wnt6, Wnt9a and Wnt5b (1µg/ml) were directly added in the organoid culture. As demonstrated by the histograms organoid survival following radiation was improved with treatment of WNT ligands WNT5a (*, $p<0.007$), WNT6 (*, $p<0.003$) and WNT9a (*, $p<0.008$) (unpaired *t*-test, two-tailed). compared to irradiated control.

B. Immunoblot to detect Rspondin1 in WT and Porcn null BMMΦ CM. Presence of Rspondin1 was noted in both WT and Porcn null BMMΦ CM indicating that deletion of *Porcn* in BMMΦ does not modulate Rspondin1 expression.

Supplementary Figure 8: Un-cropped Western Blot images from Figure 5B, Supplementary Figure 4A and Supplementary Figure 7B

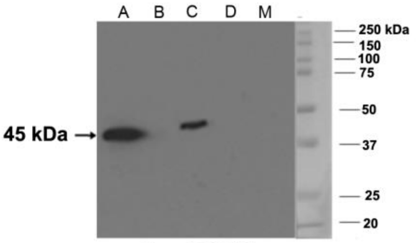

Figure 5B TSG101

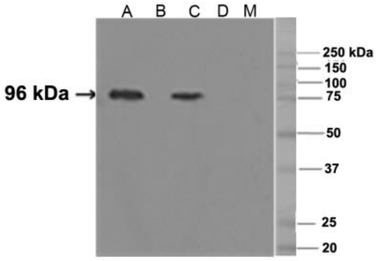

Figure 5B ALIX

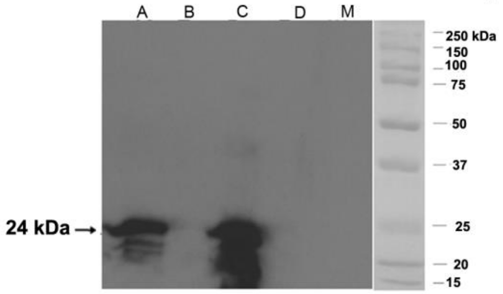

Figure 5B CD9

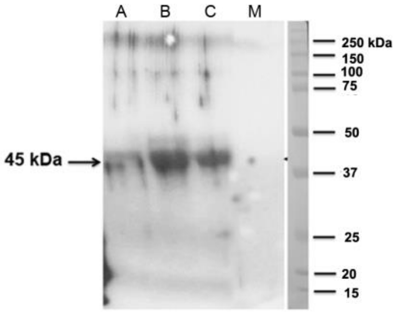

Supplementary Figure 4A TSG101

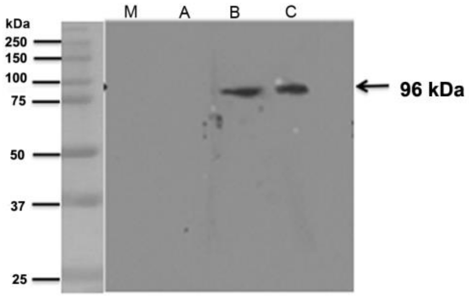

Supplementary Figure 4A ALIX

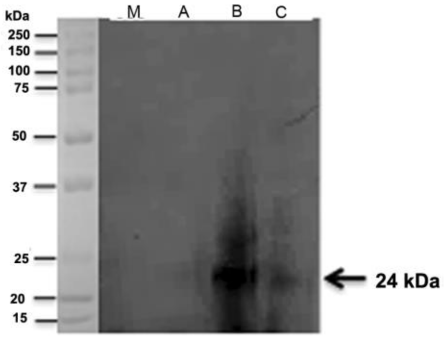

Supplementary Figure 4A CD9

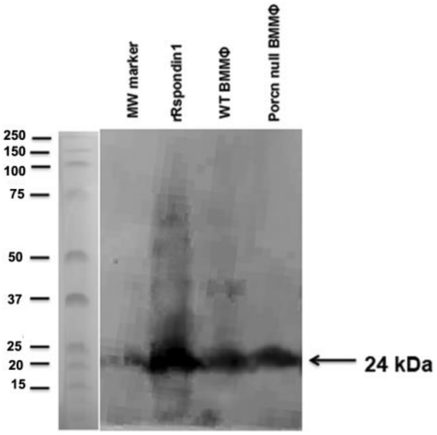

Supplementary Figure 7B

Figure 5B

M = MW Marker

A= WT BMMΦ CM EV
B= WT BMMΦ CM Effluent
C= *Porcn* null BMMΦ CM EV
D= Porcn null BMMΦ CM Effluent

Supplement Figure 4A

A= Exosome Depleted WT BMMΦ CM
B = Purified Exosome
C = WT BMMΦ CM
M = MW Marker
